# Engineering human cell spheroids to model embryonic tissue fusion *in vitro*

**DOI:** 10.1371/journal.pone.0184155

**Published:** 2017-09-12

**Authors:** David G. Belair, Cynthia J. Wolf, Carmen Wood, Hongzu Ren, Rachel Grindstaff, William Padgett, Adam Swank, Denise MacMillan, Anna Fisher, Witold Winnik, Barbara D. Abbott

**Affiliations:** 1 Toxicity Assessment Division, US EPA, Office of Research and Development, National Health and Environmental Effects Research Laboratory, Research Triangle Park, North Carolina, United States of America; 2 Research Cores Unit, US EPA, Office of Research and Development, National Health and Environmental Effects Research Laboratory, Research Triangle Park, North Carolina, United States of America; Instituto Butantan, BRAZIL

## Abstract

Epithelial-mesenchymal interactions drive embryonic fusion events during development, and perturbations of these interactions can result in birth defects. Cleft palate and neural tube defects can result from genetic defects or environmental exposures during development, yet very little is known about the effect of chemical exposures on fusion events during human development because of a lack of relevant and robust human *in vitro* assays of developmental fusion behavior. Given the etiology and prevalence of cleft palate and the relatively simple architecture and composition of the embryonic palate, we sought to develop a three-dimensional culture system that mimics the embryonic palate and could be used to study fusion behavior *in vitro* using human cells. We engineered size-controlled human Wharton’s Jelly stromal cell (HWJSC) spheroids and established that 7 days of culture in osteogenesis differentiation medium was sufficient to promote an osteogenic phenotype consistent with embryonic palatal mesenchyme. HWJSC spheroids supported the attachment of human epidermal keratinocyte progenitor cells (HPEKp) on the outer spheroid surface likely through deposition of collagens I and IV, fibronectin, and laminin by mesenchymal spheroids. HWJSC spheroids coated in HPEKp cells exhibited fusion behavior in culture, as indicated by the removal of epithelial cells from the seams between spheroids, that was dependent on epidermal growth factor signaling and fibroblast growth factor signaling in agreement with palate fusion literature. The method described here may broadly apply to the generation of three-dimensional epithelial-mesenchymal co-cultures to study developmental fusion events in a format that is amenable to predictive toxicology applications.

## Introduction

Morphogenetic fusion and tissue patterning during embryonic development is governed by reciprocal epithelial-mesenchymal interactions and cell-matrix interactions[1] and involves complex biophysical and molecular processes[2,3]. For example, in the developing embryo, segmentation of the cardiac tube and formation of the cardiac septum and valves are driven by interactions between the endocardium and myocardium[1] with a contribution from the epicardium, which also promotes the formation of coronary vasculature[4]. Non-neural ectoderm, neural ectoderm, presumptive neural crest cells, and mesodermal cells coordinate invagination of the neural plate and ultimately fusion of the neural tube[1]. Patterning and fusion of the secondary palate is coordinated by peridermal cells, neural crest-derived palatal mesenchyme, and medial edge epithelium[1,5]. Disruptions of the biophysical or molecular events of fusion by genetic mutations, chemical exposures, or a combination of both can result in heart defects, neural tube defects, and cleft palate, respectively[1,3]. The study of developmental tissue fusion requires *in vivo* or *ex vivo* rodent studies to characterize the cellular and molecular mechanisms of fusion and identify putative teratogenic chemicals. However, the lack of robust *in vitro* models for human embryonic tissue morphogenesis motivates the engineering of culture models that mimic the architecture of embryonic tissues and are suitable for modeling complex fusion events.

Cleft palate occurs upon failure of the palatal shelves to properly elevate, adhere, or fuse. Cleft palate can be caused by genetic or environmental factors[6] and is the most common craniofacial defect, affecting nearly 0.1% of live births globally[7]. The limited throughput and limited human relevance of animal models of palate fusion constrains our ability to identify and characterize human cleft palate teratogens. Embryonic palatal tissue is relatively simple in that it consists of a naïve epithelium surrounding palatal mesenchyme, and palatal fusion serves as a model system to design fusion-competent engineered tissues *in vitro*. We sought to engineer a model system that mimics the key architectural, phenotypic, and functional characteristics of palatal fusion *in vitro* using human cells for toxicity assessment applications.

Palate fusion initially involves the elevation of apposing palatal shelves *via* mesenchymal proliferation and extracellular matrix deposition mediated by fibroblast growth factor (FGF) and sonic hedgehog (SHH) signaling between palatal epithelium and mesenchyme[8]. Palatal shelf extension is accompanied by removal of the periderm layer surrounding the medial edge of apposing palates, a process mediated by transforming growth factor β3 (TGFβ3) signaling [9], leaving the basal medial edge epithelial (MEE) cells exposed on either palatal shelf. Adhesion of the palatal shelves at the mid-line[10] initiates a cascade of events involving breakdown of the basement membrane, removal of the MEE cells from the seam between apposing palatal shelves, and biophysical coalescence of the palatal mesenchyme regulated by signaling through pathways including TGFβ [11] and epidermal growth factor (EGF) [12]. The key cellular features of palate fusion are the removal of the MEE cells from the palatal mid-line seam and the formation of a confluent mesenchymal shelf. Given the extensive crosstalk between mesenchymal and epithelial cells in the palate, it is necessary to develop a model *in vitro* system that can mimic the epithelial-mesenchymal interactions in the palate.

Bioengineering strategies have been used to promote epithelial-mesenchymal interactions in a variety of tissue types, but existing strategies are not amenable to developing robust, three-dimensional, fusion-competent microtissues. The osteogenic phenotype of embryonic palate requires that engineered palate-like tissues be capable of differentiating toward an osteogenic lineage[13]. However, epithelial tissue engineering approaches[14] commonly use fibroblasts or multipotent stromal cells to recapitulate the mesenchymal compartment of epithelial tissues. Furthermore, palatal MEE is immature and non-keratinized, and thus epithelial-mesenchymal co-cultures should be generated with an immature epithelial cell type in submerged culture to prevent keratinization and epithelial differentiation. We established a three-dimensional co-culture model of multipotent human Wharton’s jelly stromal cell (HWJSC) spheroids that were first differentiated down the osteogenic lineage and subsequently seeded on the outer spheroid surface with human primary epidermal keratinocyte progenitor cells (HPEKps). Co-cultured HWJSC/HPEKp spheroids exhibited fusion behavior in culture, indicated by removal of HPEKp from adjacent contacting spheroids, and fusion was dependent upon EGF signaling and FGF signaling in agreement with palate fusion literature. This 3D co-culture model exhibits an osteogenic phenotype characteristic of embryonic palatal tissue and is amenable to evaluating chemicals for their potential to interfere with the molecular and cellular regulation of tissue fusion. The method used here will be broadly applicable for predictive toxicology applications and for studying epithelial-mesenchymal interactions and engineered tissue fusion of other developmental fusion processes.

## Materials and methods

### Cell culture

Primary HWJSCs (ATCC) were thawed, plated on CellBIND tissue culture flasks (Corning), and expanded in HWJSC growth medium (GM) containing Mesenchymal Stem Cell Basal Medium (ATCC PCS-500-030) and Mesenchymal Stem Cell Growth Kit for Adipose and Umbilical-derived MSCs—Low Serum (ATCC PCS-500-040). HWJSCs were passaged at 70–80% confluence by washing flasks in Dulbecco’s phosphate buffered saline (DPBS; ATCC), dissociating using Trypsin (ATCC) and Trypsin Neutralizer (ATCC), and counting by the Trypan blue dye exclusion method and Countess Automated Cell Counter (Invitrogen). HWJSCs were refrozen using freezing medium consisting of GM with 10% DMSO (Fisher). For all experiments, HWJSCs were thawed from the secondary bank of cells, passaged with Trypsin and Trypsin Neutralizer, counted, and either re-plated or used between passage 4–9 in experiments.

Primary HPEKp (CellnTec) were thawed, plated on CellBIND tissue culture flasks, and expanded in epithelial cell growth medium, CnT-PR (CellnTec). HPEKp were passaged at 70–80% confluence by washing flasks with DPBS, dissociating using Accutase (CellnTec), and counting using Trypan blue dye exclusion and Countess Automated Cell Counter. HPEKp were refrozen using 2X freezing medium, CnT-CRYO-50 (CellnTec), diluted in CnT-PR. HPEKp were thawed from the secondary bank of cells, passaged using Accutase, counted, and either re-plated or used between passage 4–9 in experiments.

### Generation and osteogenic differentiation of HWJSC spheroids

Silicone molds containing 300 microwells per well of a 24 well plate were generously donated from the University of Wisconsin-Madison and were generated as described elsewhere[15] using Hydrosil A+B (Siladent). Agarose microwell molds were generated using Ultra-Pure Agarose (Invitrogen) that was dissolved at 2% in DPBS, heated to boiling, and added into silicone molds at 0.4 mL/well. After solidification, agarose microwell molds were plated in 24 well tissue culture plates (BD Biosciences) with 0.5 mL GM, and the plate was centrifuged at 2,100 x g for 10 min and incubated in a humidified environment at 37°C and 5% CO_2_ (hereafter referred to as normal culture conditions) for at least 15 minutes. Before cell seeding, fresh 0.5 mL GM containing 1% antibiotic-antimycotic (10,000 units/mL penicillin, 10,000 μg/mL streptomycin, 25 μg/mL Gibco Amphotericin B; Gibco) was added to each well. HWJSCs were passaged and counted and either stained or directly plated. For experiments with HPEKp co-culture, HWJSCs were stained with 10 μM CellTracker Orange Dye (Thermo Fisher Scientific) in GM for 20 min under normal culture conditions and centrifuged at 200 x g for 5 min to remove supernatant. HWJSCs (stained or un-stained) were suspended at 0.6, 1.2, or 1.8 x 10^6^ cells/mL in GM with 1% antibiotic-antimycotic and added at 0.5 mL/well of the 24 well plate containing agarose microwell molds to generate spheroids with 1,000, 2,000, or 3,000 cells/spheroid respectively. HWJSCs were incubated in molds at room temperature for 10 min, centrifuged at 200 x g for 5 min, and incubated overnight under normal culture conditions. On the day after seeding, medium from each well was slowly removed and replaced with 0.75 mL/well of osteo-induction medium (OM) containing StemPro Osteogenesis Differentiation Kit (Gibco A1007201) with 1% antibiotic-antimycotic. HWJSCs were cultured in OM under normal culture conditions for the specified duration, with medium changes every 2–3 days.

### Alkaline phosphatase activity assay

HWJSC spheroids were assayed for alkaline phosphatase activity using Vector Red Alkaline Phosphatase Substrate Kit (SK-5100) following the manufacturer’s instructions. Briefly, HWJSC spheroids were cultured (for the specified duration), harvested, incubated in alkaline phosphatase substrate (diluted into DPBS) at room temperature on a rotisserie lab rotator for 4 hours, and fixed in 4% formaldehyde (Sigma; prepared fresh in DPBS) and 1% Triton X-100 (Sigma) for 3 hours at 4°C on a rotisserie lab rotator. Spheroids were washed in DPBS and imaged on a Nikon A1 confocal laser scanning microscope equipped with Galvano scanner, dichroic mirror for 405/488/561/640 nm wavelengths, filters for 450/50, 525/50, and 595/50 nm wavelengths, lasers for 401.4, 488, 561.8, and 638 nm wavelengths, and 10X Plan Apo objective. Mean fluorescence intensity of spheroids was calculated, and spheroids from three independent experiments were analyzed using two-way ANOVA at α = 0.05. Full details on alkaline phosphatase staining can be found in Supplementary Methods.

### Gene expression analysis by qRT-PCR

Unstained HWJSC spheroids collected at day 1, 4, 7, 14, or 21 were assayed using the TaqMan Gene Expression Cells-to-CT Kit (Thermo Fisher AM1728) following a modified protocol from the manufacturer. HWJSC spheroids were harvested, washed, lysed (with three freeze-thaw cycles and mortar-pestle homogenization), and treated with DNase using Cells-to-CT reagents and protocol. Samples were reverse transcribed following Cells-to-CT protocol, and reverse transcribed samples were amplified with PCR using TaqMan probes and master mix (Thermo Fisher) with an ABI 7900HT thermocycler. The list of primers is provided in S1 Table. Cycle threshold (C_T_) values were calculated using the ABI software using *GAPDH* as the reference gene based on its stability over time (± 2 C_T_). All samples were run in biological triplicate and normalized to the ‘Day 0’ time point using the 2^-ΔΔCT^ method. Statistical comparisons were made on normalized values relative to a mean value of ‘1’ using a two-tailed t-test at α = 0.05. A detailed procedure can be found in Supplemental Methods (S1 File).

### Cultivating and harvesting HWJSC spheroids for RNA sequencing (RNASeq)

HWJSC spheroids were prepared for RNAseq using RNeasy mini kit (Qiagen) following a modified version of the manufacturer’s protocol. Unstained HWJSC spheroids from a single well were harvested at day 1 or day 7 of culture and transferred to separate microcentrifuge tubes for each condition. For fusion experiments with RNASeq, unstained HWJSC/HPEKp or HWJSC spheroids were harvested from three wells per time point and pooled in separate microcentrifuge tubes for each condition. Spheroids were washed in DPBS, incubated in 350 μL per sample of buffer RLT containing 1% β-mercaptoethanol (Sigma), and subjected to three rounds of vortexing (1.5 min) with 1 mm zirconium beads followed by centrifugation at 12,000 x g for 5 min (at 4°C). Sample lysates were diluted 1:1 in 70% molecular biology grade ethanol (Fisher) in nuclease-free water (Gibco) and transferred to filter tubes (Qiagen). Standard RNeasy protocol was followed, including the optional DNase (Qiagen) digest for 15 min at room temperature. Purified RNA was collected in nuclease-free water and stored at -20°C. All samples were tested for RNA purity and concentration using NanoDrop and Agilent Bioanalyzer (following manufacturer’s procedure) with a cutoff of RIN>8.3 for sequencing. Samples from three independent experiments were collected and processed for RNA sequencing.

### RNA sequencing

All total RNA samples (500 ng each) were processed on Apollo324 for mRNA selection with PrepX polyA mRNA Isolation Kit (Wafergen). Library prep was carried out with PrepX mRNA 8 Protocol (Wafergen) with PCR amplification for 15 cycles. PCR products were cleaned up on Apollo324 with PCR Cleanup 8 Protocol. The volume of purified libraries was about 10 μL, and sample concentration and purity were analyzed with Qubit (Invitrogen) and Agilent Bioanalyzer, respectively. Libraries were diluted to 4 nM, which was verified by Qubit, and pooled for sequencing. Pooled libraries were denatured and diluted according to Illumina NextSeq protocols. The final concentration for sequencing was 1.8 pM + 3% Phix (Illumina sequencing control product). The sequencing data for run GRC316 are stored in Illumina BaseSpace, available at https://basespace.illumina.com/s/bq9B2OTC1QqB.

### Gene ontology and pathway analysis of transcriptomics data

Transcriptomics data were aligned to the human genome (hg19) using STAR 2.4.1d and quantified using RefSeq Transcripts 2015-08-04 in Partek Flow. Gene counts were normalized to generate reads per kilobase per million (RPKM) values for principal component analysis or were quantified using differential gene expression analysis in Partek Flow. The up- and down-regulated genes were tabulated using a cutoff of FDR step-up < 0.05 for all genes and were input to DAVID gene ontology v6.8 functional annotation clustering. A representative gene ontology (GO) term from each functional cluster was chosen (based on the highest number of genes represented by the GO term in each cluster with Bonferroni *p*-value < 0.05), and the cluster enrichment was tabulated for the top 10 clusters of up- or down-regulated genes. Alternatively, up- and down-regulated genes were input to Ingenuity Pathway Analysis (Qiagen), and activated or inhibited upstream regulators were identified using a cutoff of Activation Z-Score greater than an absolute value of 2 and a *p*-value of overlap < 0.01. The full list of identified upstream regulators with target molecules in each pathway are provided in S2 Table.

### Harvesting HWJSC spheroids for 2D differential gel electrophoresis (2D-DIGE)

2D-DIGE was performed as described previously[16,17] with some modifications described below. A detailed description of the protocol can be found in the Supplemental Methods (S1 File). Briefly, unstained HWJSC spheroids (day 1 or day 7 in culture) from three biological replicate experiments were harvested, washed in DPBS, lysed in 2% SDS, 30 mM Tris, 5 mM DTT, homogenized, and heat denatured at 95°C for 10 min. Samples were centrifuged and supernatants were collected and sonicated, centrifuged, transferred to a new centrifuge tube, and stored at -20°C. Samples were precipitated in 20% trichloroacetic acid, centrifuged, washed in 10% trichloroacetic acid, washed in acetone, re-dissolved in 7 M urea, 2 M thiourea, 30 mM Tris pH 8.5, 2% CHAPS, and 1% nonidet P-40, and stored at -20°C. Total protein concentration was determined using QuickStart Bradford (Bio-Rad). Samples were labeled with Cy-Dye DIGE Fluor Minimal Labeling Kit (GE Life Sciences). Labeled samples were mixed with rehydration buffer and loaded onto pH 3–11 nonlinear IPG strip (GE Life Sciences). IPG strips were actively rehydrated overnight and were focused for a total of 11.6 kVhrs. IPG strips were incubated in equilibration buffer containing 10 mg/mL DTT for 15 min followed by incubation in equilibration buffer with 25 mg/mL iodoacetamide. IPG strips were then loaded onto 10% Criterion Pre-cast gels (IPG+1, 1.0 mm; Bio-Rad), sealed with 0.5% agarose overlay, and run at 200V for 1 h. Gels were scanned on a PharosFX Molecular Imager System (Bio-Rad). Images were analyzed using SameSpots (TotalLab), and spots that were differentially expressed by greater than 20% fold change (day 7 relative to day 1) and ANOVA *p*-value < 0.055 were picked for mass spectrometry identification.

### Mass spectrometry to identify differentially expressed proteins in 2D-DIGE

Protein plugs were digested with Proteomics Grade Trypsin (Sigma Aldrich), desalted using P10 C18 ZipTips (Millipore), and spotted on stainless steel MALDI plate with CHCA matrix (Protea Biosciences) in 80% acetonitrile and 0.1% trifluoroacetic acid in HPLC-grade water (Fisher Scientific). Spots were analyzed using AB-SCIEX 4800 MALDI-ToF/ToF (Applied Biosystems) running 4000 Series Explorer software (AB-SCIEX). Protein identification was performed using Protein Pilot 4.0 (AB-SCIEX) running Paragon Algorithm 4.0.0.0, and peptide sequences were cross-referenced with the human (Homo sapiens) SwissProt protein database (http://expasy.org/sprot/). Mass-selected MALDI-ToF/ToF spectra acquisition was performed to further increase protein sequence coverage. The reported proteins were identified based on a minimum of two unique MS/MS peptide sequence identifications characterized by the software confidence parameter of 95–99%. Unique proteins differentially expressed at day 7 versus day 1 were reported based on identification from at least one gel out of three (S3 Table).

### Immunofluorescence staining of day 1 and 7 HWJSC spheroids

Day 1 and day 7 HWJSC spheroids were harvested with DPBS and fixed in 4% paraformaldehyde, 1% Triton X-100 (Fisher) in DPBS (pH 7.3, prepared fresh) for 4 hours rotating at 4°C. Fixed spheroids were dehydrated and rehydrated with a graded series of methanol in DPBS, blocked with 3% BSA (Sigma) in DPBS-T, and stained with a 1:100 dilution of antibodies against collagen I (Bioss bs10423R-A488), collagen IV (Bioss bs4595R-A488), laminin (Novus NB300-144AF488), fibronectin F1 (Abcam ab198933), or IgG (Abcam ab150141). Spheroids were counter-stained with Hoechst 33258 (Life Technologies), cleared using the Clear^T2^ protocol, and imaged by confocal laser scanning microscopy. Full detailed methods can be found in Supplemental Methods (S1 File).

### Generation of co-cultured HWJSC/HPEKp spheroids

HWJSC spheroids stained with CellTracker Orange were cultured for 7 days (6 days of osteogenic differentiation), harvested, washed in DPBS, suspended at 600 spheroids/mL of OM, and cultured for 2 hours in ultra-low attachment 6 well plates (Corning) under normal culture conditions on an orbital shaker (~60 RPM). HWJSC spheroids were subsequently harvested and washed in DPBS. HPEKp were passaged with Accutase, counted with Countess automated cell counter, and incubated in CnT-PR with 5 μM CellTracker Green (Thermo Fisher) for 20 min under normal culture conditions. HPEKp were centrifuged at 200 x g for 4 min, washed in DPBS once, and suspended in serum-free keratinocyte/fibroblast co-culture medium (CnT-PR-CC; CellnTec), which was supplemented with 1% antibiotic-antimycotic. HPEKp were added (at varying seeding densities) to 600 HWJSC spheroids at 100 μL/sample, and HWJSC spheroids + HPEKp were transferred to separate wells of an ultra-low attachment 96 well plate (Corning). HWJSC spheroids + HPEKp were incubated for 6 hours on an orbital shaker (~150 RPM) under normal culture conditions and were transferred to separate wells of a 6-well ultra low attachment plate with 1.15 mL of additional CnT-PR-CC (containing 1% antibiotic-antimycotic) and incubated overnight on an orbital shaker (~120 RPM) under normal culture conditions. HWJSC/HPEKp spheroids were transferred out of each well, washed with DPBS, and suspended in CnT-PR-CC (containing 1% antibiotic-antimycotic). For quantification of epithelial coverage, HWJSC/HPEKp spheroids were transferred to ultra-low attachment plates for imaging on Nikon A1 confocal laser scanning microscope (130 μm range, 5 μm step size, 10X objective). Z-stacks were processed into maximum intensity projections, and the mean area of overlap between the green (HPEKp) and red (HWJSC) channels normalized to the total mean red area was calculated for each projected image. In experiments examining the influence of HPEKP/HWJSC seeding ratio on epithelial attachment, at least 7 images were analyzed per condition from 6 separate experiments, and statistical analysis was performed using two-way ANOVA and Tukey’s multiple comparisons test at a significance level of α = 0.05. Epithelial cell coverage was assessed for each experiment, and only spheroids with >50% epithelial coverage were used for confocal experiments.

### Establishment of morphogenetic fusion model in vitro

HWJSC/HPEKp co-cultured spheroids were generated, and after 24 h of orbital shaker culture were washed to remove unattached epithelial cells and were suspended at 300 spheroids/mL in CnT-PR-CC and added to separate wells of round bottom ultra-low attachment 96 well plates at 20–30 spheroids per well. Chemicals for inhibiting FGF signaling (CH5183284), EGF signaling (Erlotinib), and TGFβ signaling (SB431542) were purchased from SelleckChem, dissolved in DMSO at 20 mM (CH5183284), 6.5 mM (Erlotinib), and 10 mM (SB431542), and stored at -20°C. Inhibitors (or DMSO control) were diluted 1:1000 into CnT-PR-CC and added to HWJSC/HPEKp spheroids on day 0 of fusion to generate concentrations of 20 μM, 6.5 μM, and 10 μM consistent with concentrations used in the literature for *in vitro* screening of CH5183284[18] and Erlotinib[19] and for *in vitro* palate fusion inhibition with SB431542[20], respectively. Alternatively, spheroids were treated at day 0 with various concentrations of recombinant human EGF (BioLegend) that was reconstituted at 10 μg/mL in sterile 1% BSA in DPBS, diluted with 1% BSA in DPBS, and added to spheroids at either 2, 10, or 50 ng/mL final concentration in CnT-PR-CC with 1% antibiotic-antimycotic, with the vehicle control containing the same dilution of 1% BSA in DPBS. Fusion was monitored each day with confocal microscopy (using 10X objective and filters for red and green wavelengths), and medium was replenished with inhibitors or EGF on day 2 of fusion. In experiments with chemical inhibitors, cellular ATP content was evaluated using CellTiter Glo 3D following the manufacturer’s protocol as follows. On fusion day 4, medium in each well was replaced with 100 μL per well of CellTiter Glo 3D reagent (Promega), and spheroids was incubated for 20 min on an orbital shaker at 37°C. Reagent from each well was then transferred to a white opaque 96 well plate (Falcon) and read on a luminescence plate reader with 10 s integration time per well (Clariostar BMG Labtech). The cellular ATP content from six replicate wells per condition was normalized to the DMSO control, and data are represented as mean relative cellular ATP ± 95% confidence interval from six replicates per condition aggregated over at least two independent experiments. One-way ANOVA with Dunnett post-hoc test was performed at α = 0.05 for statistical significance.

Fusion was assessed using morphometric analysis of a representative confocal z-slice, approximately 60 μm from the bottom of each well, using NIS Elements v4.3 (Nikon). A region of interest was drawn around each sample, outlier pixels were removed through image processing, and a threshold was applied to each image uniformly to quantify the percentage of overlap between green and red channels relative to the total red area calculated from each sample. The red/green overlap percentage was selected as representative of HPEKp co-localized within the seam between fusing HWJSC spheroids. The percent red/green overlap was calculated for each time point and sample and normalized to the day 0 time point in order to quantify the removal of HPEKp from the seam between adjacent spheroids over time. The mean normalized percent red/green overlap was calculated for three samples per condition, aggregated from at least two independent experiments. Statistical analysis was performed using two-way ANOVA and Dunnett’s multiple comparisons test at a significance level of α = 0.05.

Transcriptomics analysis was performed on fusing HWJSC/HPEKp or HWJSC spheroids generated without cell tracking dye but following the same protocol as above (except unstained HWJSC spheroids were harvested and incubated in CnT-PR-CC without epithelial cells for 24 h as a control to mirror the procedure used to coat HWJSC spheroids with HPEKp). Spheroids were added to round bottom ultra low attachment 96 well plates at 50 spheroids per well, and spheroids were harvested and pooled from three separate wells at each time point. Harvested spheroids were washed with DPBS and input to RNASeq workflow as described above. Normalized RPKM values were calculated for each sample, and samples were input to principal component analysis and plotted on three principal components.

## Results

### Generating and characterizing HWJSC spheroids

**Fig 1:**
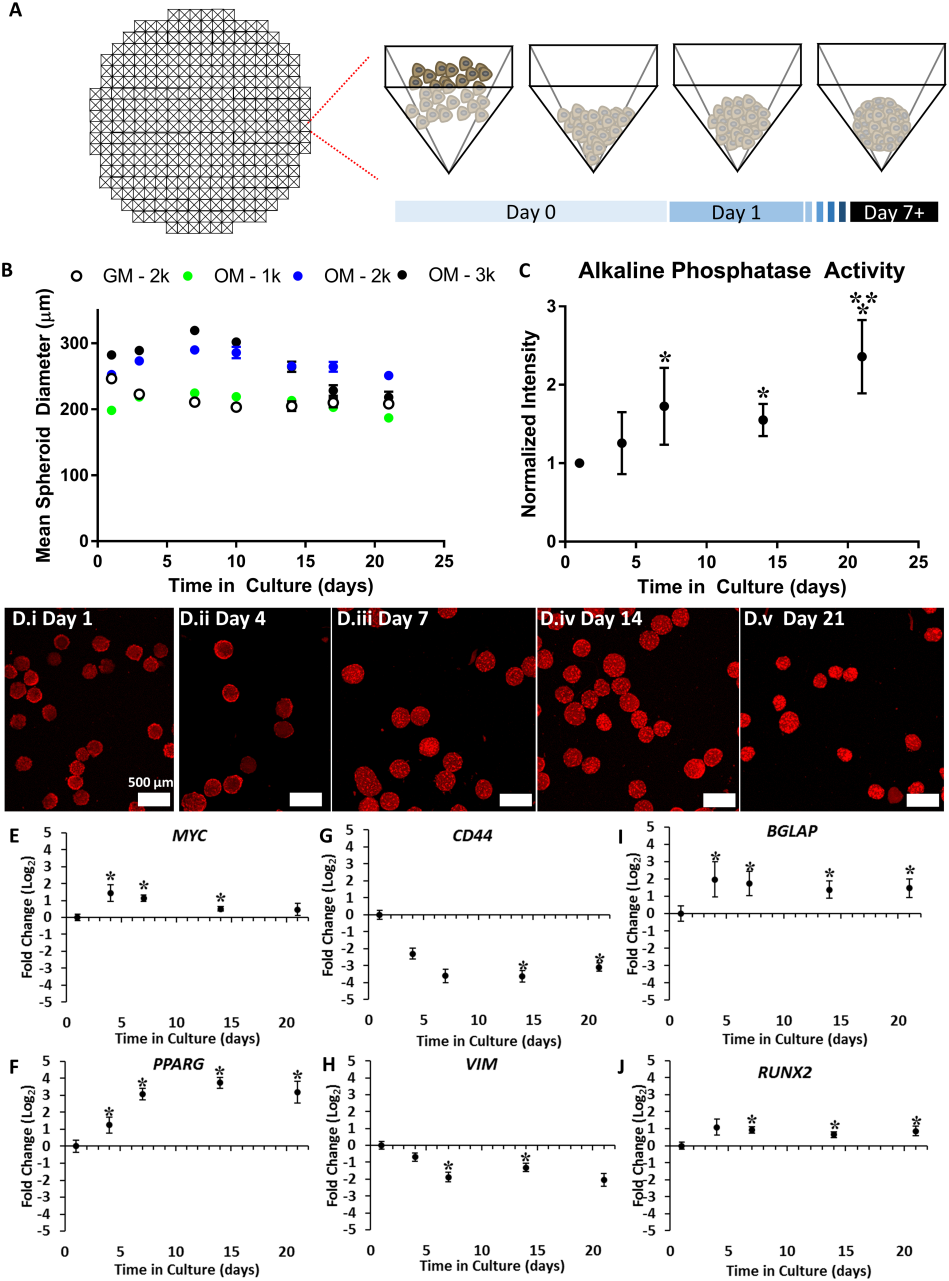
Characterization of HWJSC spheroids and time-course of osteogenic differentiation. A: Schematic of HWJSC spheroid generation in agarose microwells, generated using AggreWell 800 as a template. B: Data representing the mean spheroid diameter of HWJSC spheroids at three different initial seeding densities (1k, 2k, 3k HWJSCs per spheroid) and in either osteogenic differentiation medium (OM) or growth medium (GM). HWJSCs were seeded in GM on day 0 and were subsequently cultured at day 1 and beyond in either OM or GM for the remainder of the culture (with medium changes every 2–3 days). HWJSC spheroid mean diameter ± 95% confidence interval was calculated for 40 spheroids per condition per time point from one representative experiment. C: Quantified mean intensity of 2k HWJSC spheroids cultured in OM and stained using an alkaline phosphatase substrate kit. Data are presented as the mean fluorescence intensity ± SEM for three independent experiments, normalized to the day 1 time point. Asterisks denote statistical significance calculated on raw mean intensity values at α = 0.05 of each condition relative to the day 1 and day 4 time points (*) or relative to the day 7 and day 14 time points (**) using two-way ANOVA and Tukey’s post-hoc test. D: Fluorescent micrographs representing maximum intensity projections of HWJSC spheroids that were stained for alkaline phosphatase activity at the indicated time point after the initiation of spheroid culture. Scale bar represents 200 μm. E-J: Fold change in gene expression over time of 2k HWJSC spheroids cultured in OM. Data represent fold change relative to GAPDH housekeeping gene and the day 1time point for MYC (E), PPARG (F), CD44 (G), VIM (H), BGLAP (I), and RUNX2 (J). Asterisks denote statistical significance relative to a mean value of ‘1’ using a one-tailed Student’s t-test (α = 0.05) for three independent experiments.

Developmental morphogenetic fusion involves signaling crosstalk between mesenchymal cells and epithelial cells that are separated by a basement membrane. We developed a co-culture model using human mesenchymal and epithelial cells that could be used to assess *in vitro* tissue fusion behavior. Engineered tissues were designed to resemble the cellular composition and structure of the embryonic palate due to the simple architecture of the palate and the need for an *in vitro* model of palate fusion. The mesenchymal compartment of the embryonic palate consists of cranial neural crest-derived mesenchyme embedded in a vascularized ECM. Palatal mesenchyme exhibits an osteogenic phenotype (characterized by elevated *Runx2*, *Alpl*, and *Col1a1* expression in mouse studies[21]) prior to fusion that supports the eventual mineralization and intramembranous ossification of the fused secondary palate. Given the three-dimensional (3D) structure and osteogenic phenotype of the palate and motivated by previous studies showing that 3D mesenchymal cell spheroids potentiate osteogenesis *in vitro*[22,23], we generated 3D mesenchymal cell spheroids and differentiated them down the osteogenic lineage to mimic palatal mesenchyme. Mesenchymal cell spheroids here were composed of HWJSCs (Fig 1A) that were seeded at three densities (1,000, 2,000, and 3,000 HWJSCs/spheroid) and cultured in osteo-induction medium (OM) for 21 days (2,000 cell HWJSC spheroids were cultured in growth medium, GM, as a control). Initial HWJSC cell seeding density significantly influenced the mean spheroid diameter, and all three spheroid sizes that were cultured in OM exhibited increasing diameter between days 1–7 and decreasing diameter between days 7–21 (Fig 1B). The 2,000 cell HWJSC spheroids cultured in GM exhibited decreasing diameter throughout the 21-day culture that was accompanied with cell debris accumulation over time (S1 Fig). Similar debris accumulation was observed in the 3,000 cell HWJSC spheroids that were cultured in OM between days 14–21, which suggests cell death in this condition. Therefore, 2,000 cell HWJSC spheroids were selected for further experiments.

We characterized the osteogenic phenotype of HWJSC spheroids by examining alkaline phosphatase activity and gene expression for stemness, mesenchymal, and osteogenic genes. HWJSC spheroids were cultured in GM for one day, induced with OM, and treated with an alkaline phosphatase fluorescent substrate at the end of each time point. HWJSCs exhibited increased mean fluorescence at days 7, 14, and 21 of culture, indicating increased alkaline phosphatase activity at these time points relative to the day 1 control and relative to day 4 (Fig 1C and 1D). Alkaline phosphatase activity at day 21 of differentiation was higher than all other time points, while no increase in activity was observed at day 4 relative to day 1 (Fig 1C and 1D). We assessed HWJSC phenotype over time in culture by monitoring pluripotency markers, mesenchymal markers, osteogenesis markers, and a putative adipogenesis marker as a negative control. The putative pluripotency marker, *MYC*, was significantly increased at days 4, 7, and 14 of culture in OM versus the day 1 control (Fig 1E). The mesenchymal genes *CD44* (Fig 1G) and *VIM* (Fig 1H) were significantly decreased at day 7 and 14 and at day 14 and 21 of spheroid culture in OM, respectively. The osteogenic genes *BGLAP* (Fig 1I) and *RUNX2* (Fig 1J) were significantly upregulated at day 4 and beyond and at day 7 and beyond, respectively. We did not observe a significant fold change at any time point of the housekeeping gene, *ACTB* (Figure A in S2 Fig), the pluripotency genes *NANOG* (Figure B in S2 Fig), *SOX2* (Figure C in S2 Fig), *KLF4* (Figure D in S2 Fig), or *POU5F1* (Figure E in S2 Fig), the mesenchymal gene *ENG* (Figure F in S2 Fig), or the osteogenesis-related genes *ALPL* (Figure G in S2 Fig) or *SPP1* (Figure H in S2 Fig). Finally, we observed a significant increase in the adipogenic control gene, *PPARG*, at day 4 and beyond (Fig 1F), which may suggest that at least some of the HWJSCs underwent adipogenic differentiation in osteo-induction medium. We addressed this hypothesis in subsequent transcriptomic and proteomic analyses of HWJSC spheroids. Our data demonstrate that 7 days of osteogenic differentiation was sufficient to induce gene expression changes and elicit increased alkaline phosphatase activity consistent with osteogenic differentiation.

**Fig 2:**
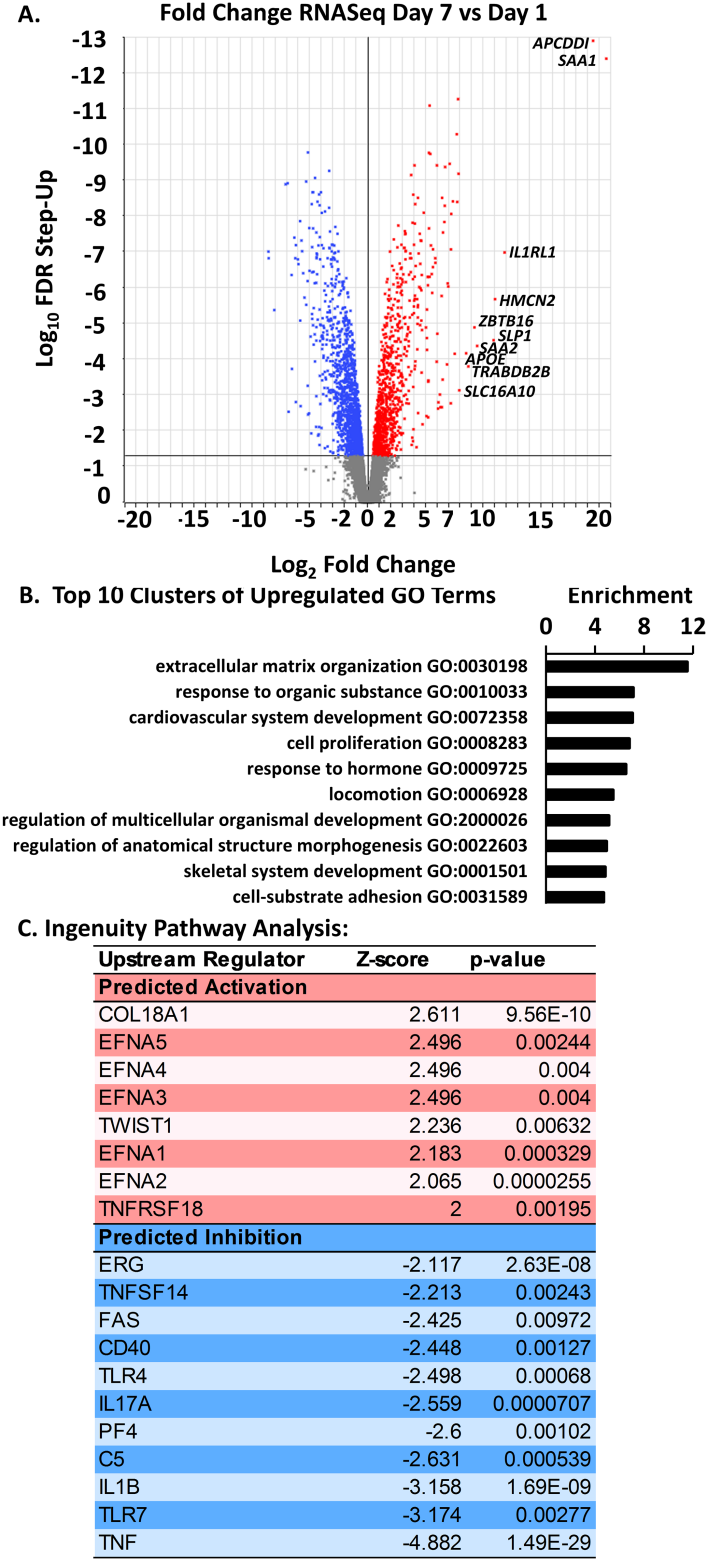
Transcriptomic profile of HWJSC spheroids upon osteogenic differentiation. A: Volcano plot of FDR step-up (log-10) versus expression fold change (log-2) of differentially expressed genes comparing day 7 to day 1 HWJSC spheroids. A cutoff of FDR step-up < 0.05 was selected for downstream analysis of gene ontology. The top 10 up-regulated genes (in terms of magnitude of fold change via RNASeq) are transcribed on the figure. B: Gene ontology (GO) analysis of HWJSC spheroids cultured for 1 d or 7 d (osteogenic differentiation for 6 d). GO analysis was performed on osteogenic HWJSC spheroids (7 d) relative to 1 d spheroids using only genes that were upregulated relative to 1 d spheroids with FDR step-up < 0.05. Enrichment scores were derived from GO analysis and functional annotation clustering in DAVID, and a representative GO term (with the highest number of upregulated genes in each cluster and a Bonferoni p-value < 0.05) from each cluster was chosen from the top 10 enriched clusters. C: Ingenuity Pathway Analysis of up-regulated genes at day 7 versus day 1.

We further characterized the phenotype of differentiated HWJSC spheroids using transcriptomic and proteomic analyses. We compared the transcriptomic profile of three biological replicates of day 7 versus day 1 HWJSC spheroids using RNA sequencing (RNASeq) and differential gene expression analysis. HWJSC spheroids at day 7 exhibited 1,012 significantly up-regulated and 1,544 significantly down-regulated genes compared to day 1 (Fig 2A). Analysis of three principal components generated from the reads per kilobase of transcript per million mapped reads (RPKM) values for all 11,619 mapped genes represented 97.7% of the variability in RPKM values. The day 1 and day 7 spheroid samples formed separate clusters of data points when plotted on three principal component axes (Figure A in S3 Fig), indicating a high degree of sample-to-sample reproducibility of the data set. We compared the RNASeq results to the PCR data and observed close agreement (Pearson correlation coefficient of 0.9884) in the direction and magnitude of fold changes, specifically in the statistically significant up-regulation of *MYC*, *RUNX2*, and *PPARG*, and in the significant down-regulation of *VIM* at day 7 of differentiation (Figure B in S3 Fig). Gene ontology analysis (using David v6.8) of the upregulated genes at day 7 identified functional clusters of enriched genes represented by the gene ontology terms for extracellular matrix organization, system development (cardiovascular and skeletal), response to organic substance (and response to hormone), regulation of organismal development and anatomical structure morphogenesis, and cell proliferation, locomotion, and cell-substrate adhesion (Fig 2B). Down-regulated genes at day 7 were clustered in a similar way (Figure C in S3 Fig). We input both up- and down-regulated genes into Ingenuity Pathway Analysis and observed a predicted activation of the upstream regulators COL18A1, EFNA 1–5, TWIST1, and TNFRSF18 and a predicted inhibition of upstream regulators ERG, TNFSF14, FAS, CD40, TLR4, IL17A, PF4, C5, IL1B, TLR7, and TNF in day 7 spheroids relative to day 1 (Fig 2C). Activation of TWIST1 could indicate osteoblast differentiation, and activation of COL18A1 and Ephrin A1 pathways could indicate cell signaling related to angiogenesis and cell adhesion according to gene ontology.

**Fig 3:**
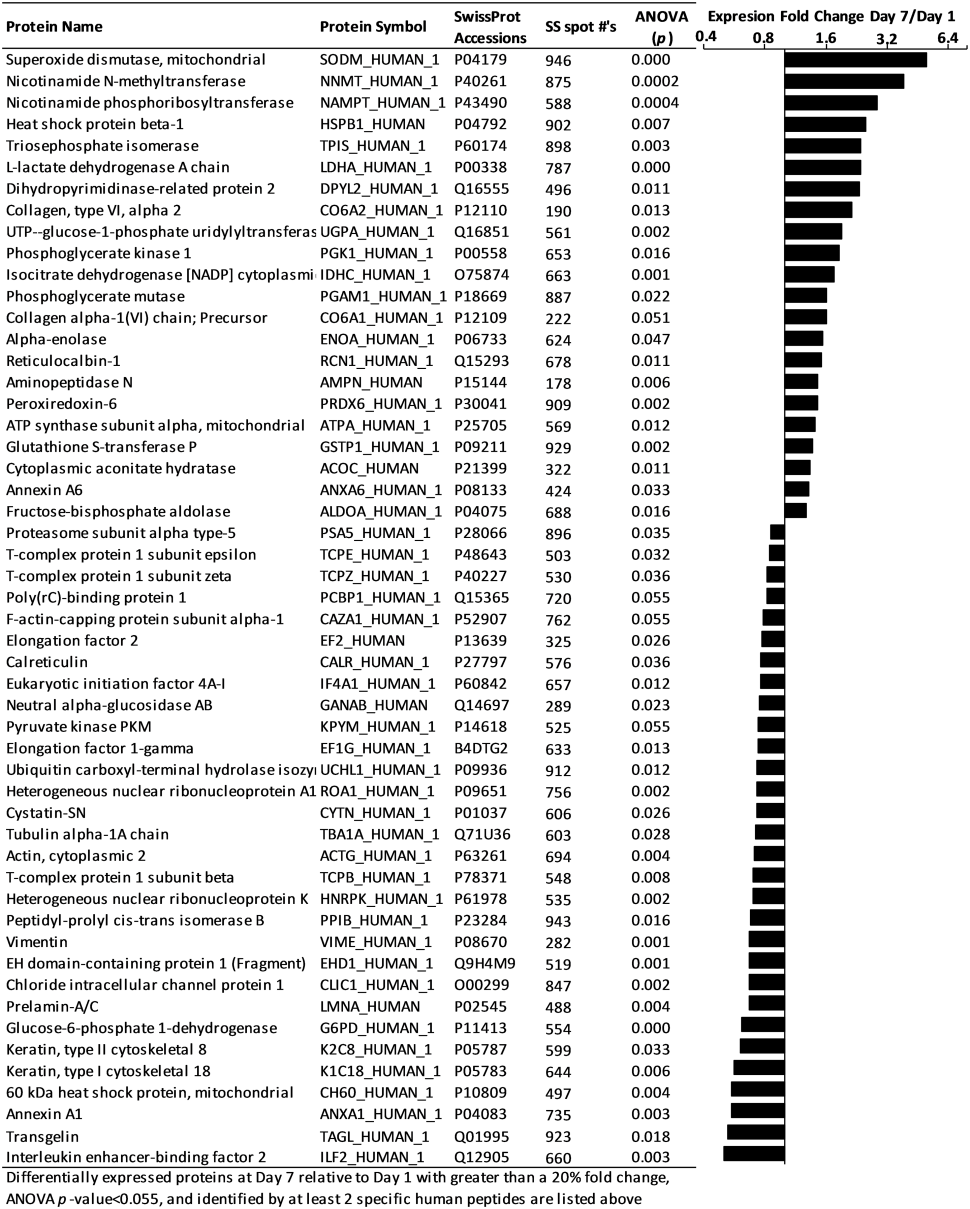
2D-DIGE analysis of osteogenic differentiation of HWJSC spheroids at day 7 versus day 1. HWJSC spheroids were cultured for either 1 or 7 days, and 2D-differential gel electrophoresis and mass spectrometry was performed to identify the differentially expressed proteins that were present on at least one gel (out of a total of 3 gels, representing randomized samples from 3 independent experiments) and identified with at least 2 peptides. Fold change was calculated for each unique spot, with an ANOVA p-value cutoff of 0.055. The protein name, symbol, SwissProt Accession, SameSpots #, and ANOVA p-value are provided in columns for each unique identified protein, and protein expression fold change in day 7 versus day 1 samples is presented as a bar graph centered around a fold change of 1 (representing no difference between day 1 and day 7).

We interrogated protein-level expression changes during HWJSC osteogenic differentiation to identify signaling molecules or extracellular matrix molecules that could inform the differentiation status of HWJSC spheroids. We performed 2D-DIGE on spheroid lysates from day 1 and day 7 spheroids and used MALDI-ToF/ToF to identify the differentially expressed genes. 2D-DIGE analysis revealed 52 differentially expressed proteins in the day 7 spheroids versus the day 1 spheroids, of which 22 unique proteins were upregulated at day 7 (Fig 3). Only 12 of the differentially expressed proteins were also differentially expressed *via* RNASeq, and these 12 comparisons between protein- and transcript-level fold change (day 7 relative to day 1) were closely correlated with Pearson correlation coefficient of 0.8560 (Figure A in S4 Fig). A similar comparison between protein fold change (*p*<0.055) and transcript fold change (with no significance threshold) of all 52 protein species yielded a Pearson correlation coefficient of 0.5285 (Figure B in S4 Fig). These data suggest that insignificant transcript-level fold changes yielded significant protein-level expression changes that were not well-correlated to the direction or magnitude of the transcript changes, which highlights post-transcriptional control over the protein expression of the majority of the 52 differentially expressed proteins. The protein- and transcript-level fold changes from day 1 to day 7 agree in the upregulation of *NNMT*, *NAMPT*, *DPYSL2*, *UGP2*, and *ANXA6* and the downregulation of *VIM*, *UCHL1*, *EHD1*, and *G6PD*, while the fold change of *ANXA1*, *ACO1*, and *COL6A1* gene expression was in the opposite direction of protein expression fold change. The differentially expressed proteins in day 7 versus day 1 HWJSC spheroids were analyzed using IPA and were associated primarily with upregulation of canonical IPA pathways including glycolysis, gluconeogenesis, sucrose degradation, glutathione redox reactions, and NRF2-mediated oxidative stress response (S4 Table). Three upregulated proteins were specifically related to the extracellular space (CO6A1, CO6A2, NAMPT). CO6A1 is listed the gene ontology for osteoblast differentiation while NAMPT may be associated with osteoblast differentiation based on recent literature[24,25]. We did not observe any overlap between the differentially expressed proteins here and the gene ontology terms for fat cell differentiation or chondrogenic differentiation, and thus we conclude that HWJSC spheroid differentiation was primarily down the osteogenic lineage.

**Fig 4:**
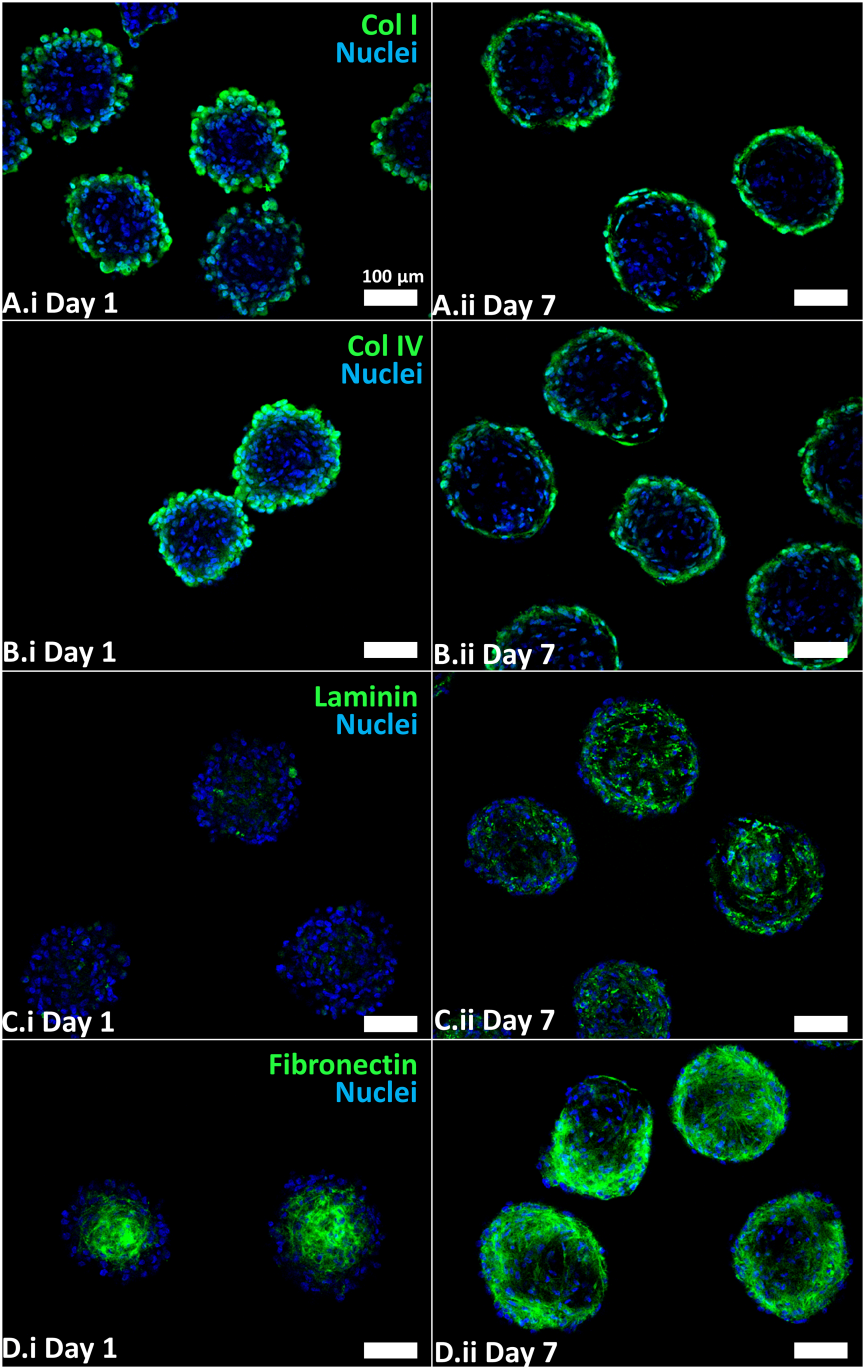
Immunofluorescence staining for extracellular matrix proteins in day 1 and day 7 HWJSC spheroids. HWJSC spheroids were cultured for either 1 day or 7 days (with 6 days of osteogenic differentiation), fixed, and stained for extracellular matrix proteins: collagen I (A) collagen IV (B), laminin (C), and fibronectin (D) and counter-staining with Hoechst. Scale bars represent 100 μm.

The epithelial and mesenchymal components of the embryonic palate are separated by a basement membrane matrix that facilitates their interaction. Given the primary goal of promoting mesenchymal-epithelial interactions in engineered palate organoids, we characterized the extracellular matrix composition of differentiated HWJSC spheroids using immunofluorescence staining. Immunofluorescence staining indicated the presence of typical basement membrane proteins including collagen I (Fig 4A), collagen IV (Fig 4B), laminin (Fig 4C), and fibronectin (Fig 4D) in both day 1 and day 7 spheroids, consistent with literature describing the ECM composition of HWJSC spheroids[26]. Collagen I and IV staining was primarily localized to the exterior of HWJSC spheroids at day 1 and day 7. Laminin staining appeared to be more pronounced in day 7 spheroids relative to day 1 spheroids and was localized throughout the spheroid interior on day 7, suggesting that osteogenic differentiation was associated with increased laminin protein deposition. Laminin genes *LAMA2*, *LAMA3*, *LAMB1*, and *LAMC3* were upregulated while *LAMA4*, *LAMA5*, *LAMB3*, and *LAMC2* genes were downregulated during differentiation (S5 Table). Furthermore, the staining pattern of fibronectin was distinctly different in day 1 versus day 7 spheroids, wherein the fibronectin staining was primarily localized to the center of HWJSC spheroids at day 1 and was more prominent in the HWJSC spheroid exterior at day 7. The fibronectin gene, *FN1*, was the most abundant transcript (in number of total reads) observed by RNASeq (S5 Table), though we observed no differences in transcript expression from day 1 to day 7, which suggests that the difference in staining pattern from day 1 to day 7 was likely mediated by matrix remodeling and post-translational changes in expression. The presence of basement membrane proteins on the surface or throughout the day 7 HWJSC spheroids may support the attachment of epithelial cells seeded on the exterior. The presence of basement membrane proteins and osteogenic phenotype in day 7 HWJSC spheroids indicates their suitability for modeling embryonic palatal mesenchyme.

### Generating co-cultured spheroids of epithelial and mesenchymal stem cells

**Fig 5:**
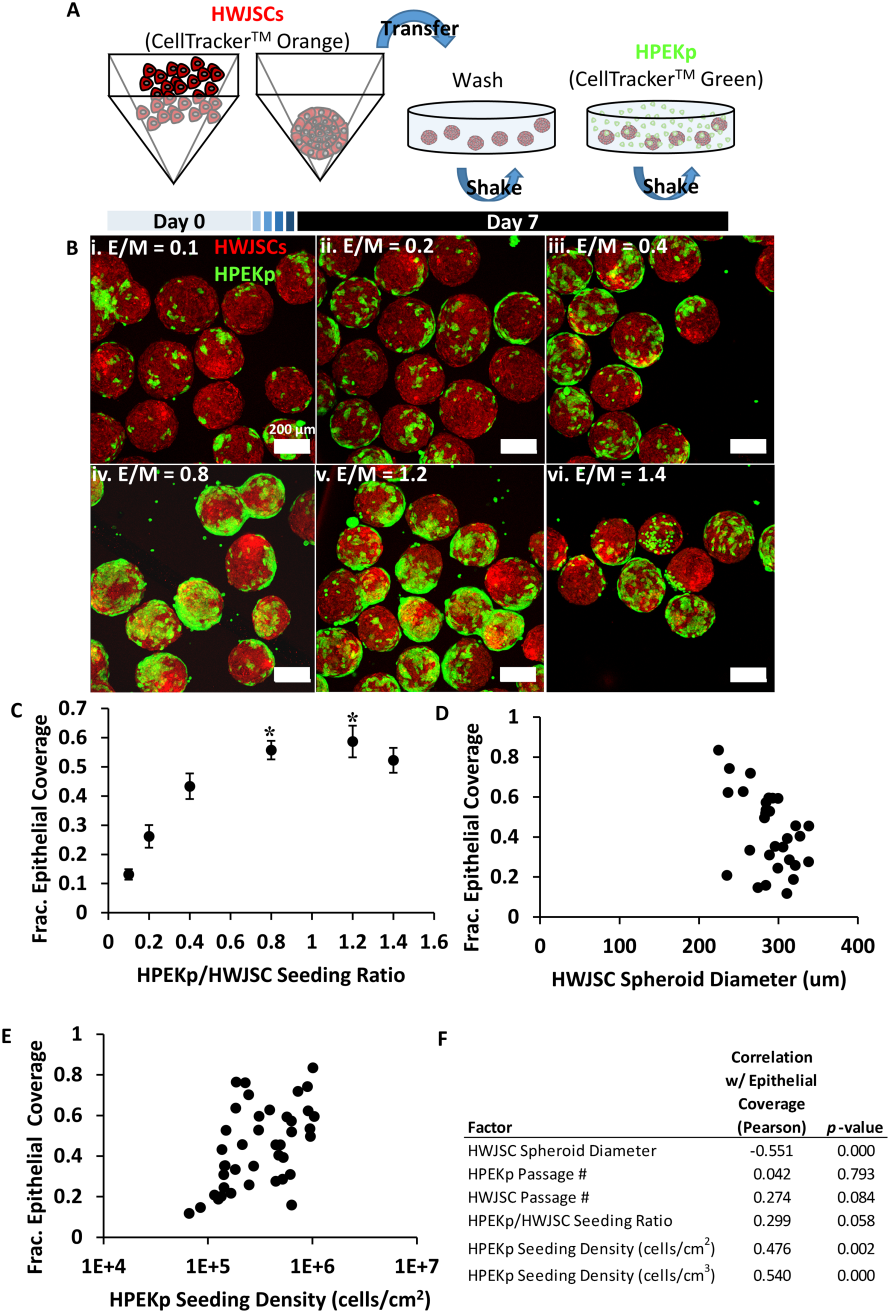
Generation of co-cultured spheroids consisting of osteogenic HWJSC spheroids seeded with epithelial cells. A: Schematic of HWJSC staining with CellTracker Orange, spheroid generation and osteogenic differentiation (7 d), washing in OM, and coating with CellTracker Green-stained HPEKp epithelial cells. B: Representative fluorescent maximum intensity projections (MaxIP) of HPEKp seeded on HWJSC spheroids after one day of incubation at epithelial/mesenchymal ratios (E/M) of 0.1 (i), 0.2 (ii), 0.4 (iii), 0.8 (iv), 1.2 (v), and 1.4 (vi). C: Quantified HPEKp/HWJSC co-localization after one day of incubation at E/M ratios from 0.1–1.4, presented as mean ± 95% confidence interval of fractional epithelial coverage represented by 7 confocal z-stacks per condition and a total of 6 independent experiments. Asterisks represent statistical significance with one-way ANOVA and Tukey’s post-hoc test of each condition relative to E/M = 0.1, 0.2, and 0.4. All conditions were statistically higher than E/M = 0.1 and 0.2. Mean epithelial coverage was statistically indistinguishable between E/M = 0.8, 1.2, and 1.4, and E/M = 1.4 was indistinguishable from E/M = 0.4. D: Quantified HPEKp/HWJSC co-localization versus mean HWJSC spheroid diameter, presented as mean fractional epithelial coverage represented by at least one confocal z-stack per data point. E: Quantified HPEKp/HWJSC co-localization versus mean HPEKp seeding density in cells/cm^2^ of HWJSC spheroid surface area, calculated from mean HWJSC spheroid diameter in each image, presented as mean fractional epithelial coverage represented by at least one confocal z-stack per data point. F: Regression analysis, presented with a Pearson correlation coefficient and p-value for each variable, of 6 independent variables versus epithelial coverage: HWJSC spheroid diameter, HPEKp passage #, HWJSC passage #, HPEKp/HWJSC seeding ratio, HPEKp seeding density (cells/cm^2^), and HPEKp seeding density (cells/cm^3^).

We next established a 3D co-culture system, comprised of osteogenic HWJSC spheroids with human primary epidermal keratinocyte precursor cells (HPEKp) seeded on the exterior spheroid surface, to mimic the architecture of embryonic palate. HWJSC spheroids (at day 7) were incubated with HPEKp cells at varying ratios of HPEKp/HWJSC (Fig 5A), and the degree of epithelial attachment was quantified using confocal microscopy and automated image analysis of 3D maximum intensity projection images. The extent of epithelial coverage was positively correlated with the HPEKp/HWJSC seeding ratio (Fig 5B and 5C). Epithelial coverage increased up to a HPEKp/HWJSC ratio of 0.8, and there was no statistical difference in epithelial coverage between HPEKp/HWJSC ratios of 0.8, 1.2, and 1.4. Epithelial coverage on HWJSC spheroids exhibited a statistically significant negative correlation with mean HWJSC spheroid diameter (Fig 5D and 5F) and a significant positive correlation with mean HPEKp seeding density in cells per cm^2^ of HWJSC area (Fig 5E and 5F) and in cells per cm^3^ of HWJSC spheroid volume (Fig 5F). These data suggest that while HPEKp/HWJSC seeding ratio influenced epithelial coverage in controlled experiments, the correlation (indicated by the Pearson coefficient) between epithelial coverage and HWJSC spheroid size was greater in magnitude. Finally, we did not observe any significant correlation between HWJSC or HPEKp passage number and epithelial coverage (Fig 5F). All subsequent experiments were performed using HWJSC spheroids with greater than 50% surface coverage of HPEKp achieved using an HPEKp seeding density of 2 x 10^5^–9 x 10^5^ cells/cm^2^.

### Assessment and signaling perturbation of co-cultured HWJSC/HPEKp spheroid fusion

**Fig 6:**
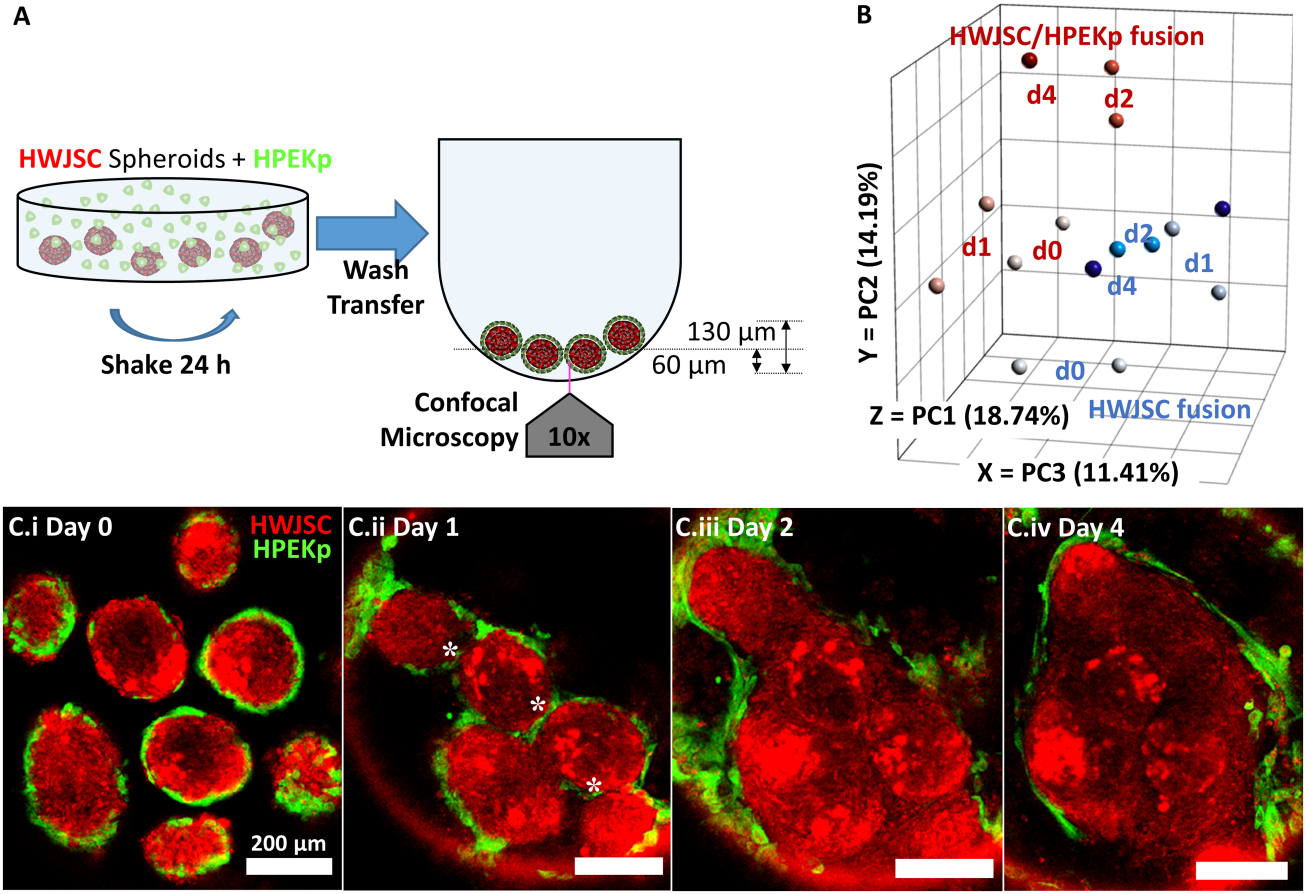
HPEKp/HWJSC spheroid fusion transcriptomics and morphometric analysis. A: Schematic of initiation of fusion experiments with HWJSC/HPEKp spheroids in ultra low attachment round-bottom plates. B: RNASeq analysis of HPEKp/HWJSC spheroid (red) or HWJSC spheroid (blue) fusion samples at day 0, 1, 2, and 4 (two samples per time point and condition, except only one sample for HPEKp/HWJSC spheroids at day 4), presented on three principal component axes of RPKM values for all samples. C: Fluorescent z-slices showing the progression of fusion of spheroids in non-adherent culture in round bottom plates. Fluorescent micrographs represent day 0 (i), day 1 (ii), day 2 (iii), and day 4 (iv) after the initiation of fusion. White asterisks denote HPEKp in the seam between adjacent spheroids. Scale bar represents 200 μm.

Co-cultured HWJSC/HPEKp spheroid fusion was assessed by culturing spheroids in non-adherent round-bottom plates (Fig 6A) and performing confocal image analysis to detect the presence of HPEKp in the seam between adjacent spheroids. During palate fusion, the MEE in the zone between adhered palatal shelves are removed over time, likely by a combination of migration, epithelial-to-mesenchymal transition, and apoptosis[6], providing a cellular benchmark with which to monitor fusion *in vitro*. On day 0 of HWJSC/HPEKp fusion, epithelial cells were observed on the exterior of HWJSC spheroids (Fig 6C.i). On day 1 of fusion, the HWJSC/HPEKp spheroids were observed in contact with one another with HPEKp still present in the zone between adjacent spheroids (Fig 6C.ii). On day 2 of fusion, HWJSC/HPEKp spheroids exhibited nearly complete fusion, as the HPEKp were restricted to the outside surface of the amalgam of HWJSC spheroids and were absent from the zones between adjacent spheroids (Fig 6C.iii). By day 4 of fusion, HWJSC/HPEKp spheroids were completely fused, as indicated by the lack of HPEKp in the seam between spheroids and the confluent HWJSC microtissue with no clear morphological characteristics of the original individual spheroids (Fig 6C.iv). We measured the coalescence of HWJSC/HPEKp spheroids and observed that the equivalent diameter of the amalgam of fusing HWJSC/HPEKp spheroids decreased by 50% between days 0–1 of fusion and remained at this diameter through days 2–4 (S5 Fig).

Transcriptomics analysis revealed distinct gene expression changes in co-cultured HWJSC/HPEKp spheroids relative to HWJSC spheroids suggesting that *in vitro* fusion involved transcriptomic changes in both epithelial and mesenchymal cells. We related the transcriptional changes of fusing HWJSC/HPEKp spheroids to that of fusing HWJSC spheroids by performing principal component analysis of the RPKM values derived from spheroid samples at days 0, 1, 2, and 4 of fusion (Fig 6B). Fusing HWJSC spheroids exhibited two main clusters of data points in all three principal components at day 0 and at days 1, 2, and 4 (Fig 6B), which suggests that most of the transcriptional changes during HWJSC spheroid fusion occurred between days 0–1. In comparison, fusing HWJSC/HPEKp spheroids exhibited three main data clusters at day 0, day 1, and days 2 and 4 (Fig 6B). The day 1 cluster from the HWJSC/HPEKp fusing spheroids was separated from the day 0 cluster primarily by PC3, while the day 2 cluster was segregated from the day 1 cluster by differences in all three principal components. The similarity in clustering between day 2 and 4 samples of HWJSC/HPEKp spheroids suggests minimal transcriptional changes between days 2–4, which agrees with our observation from confocal images that no major morphological changes occurred between days 2–4 of fusion. These data demonstrate that in the absence of epithelial cells, the transcriptional changes related to fusion occurred primarily between days 0–1, whereas in the presence of epithelial cells, the transcriptional changes occurred between days 0–1 and days 1–2, suggesting that the epithelial cells delayed the completion of fusion and promoted distinct transcriptional changes relative to fusing HWJSC spheroids.

**Fig 7:**
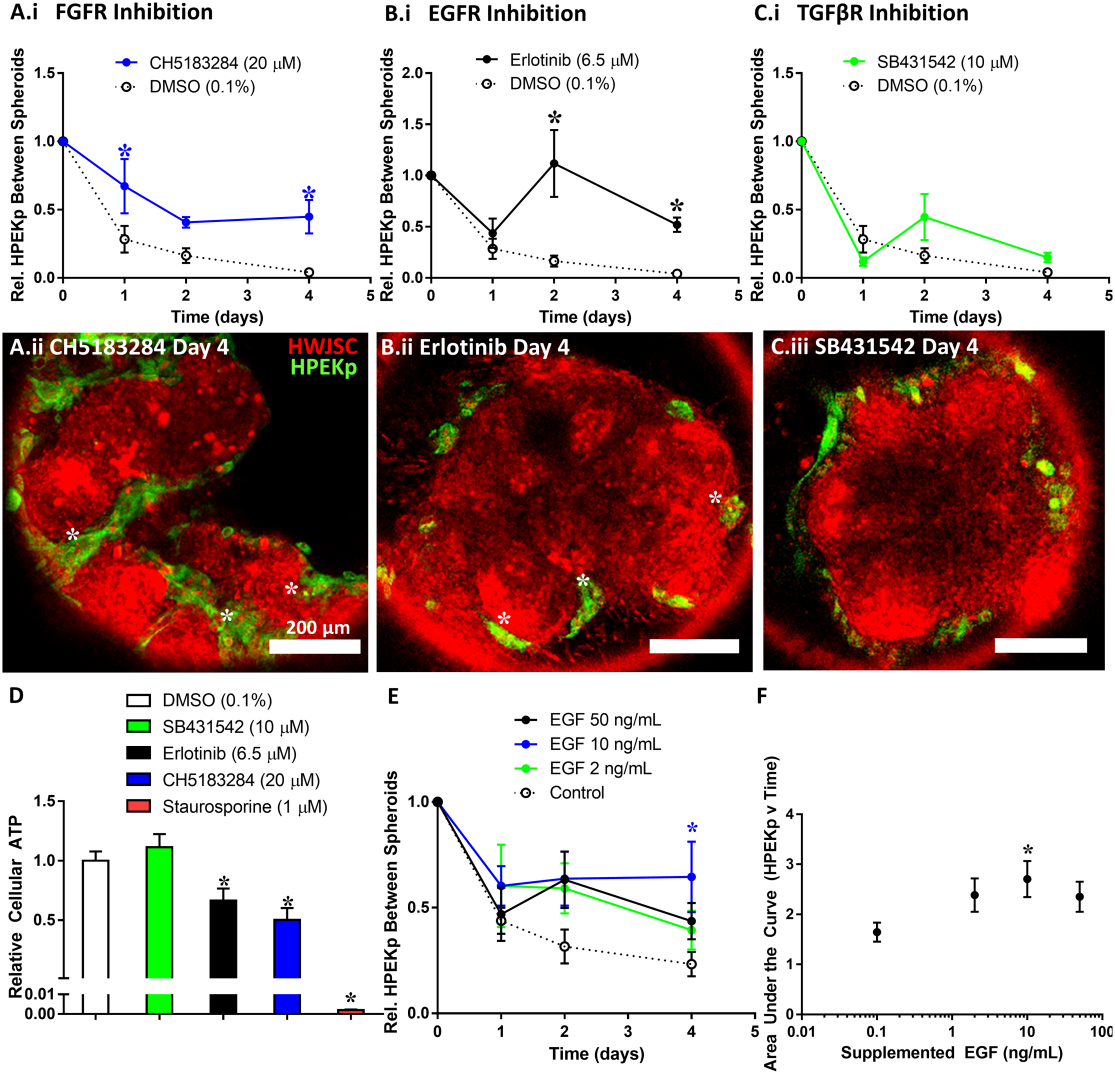
Perturbation of HPEKp/HWJSC spheroid fusion. A-C: Quantification of the relative extent of HPEKp in the seam between spheroids (a metric established to quantify the progression of fusion) with HWJSCs (mean ± SEM for 6 replicates aggregated from 2 independent experiments), normalized to the day 0 time point, upon treatment with either DMSO (0.1%), CH5183284 (20 μM) (A.i), Erlotinib (6.5 μM) (B.i), or SB431542 (10 μM) (C.i). The DMSO control is shown on all three graphs for comparison. Asterisks denote statistical significance relative to DMSO at each time point with two-way ANOVA and Dunnett post-hoc test (α = 0.05). Fluorescent micrographs represent a single representative z-plane (~55–60μm from the bottom of the amalgam of fusing spheroids) after 4 days of fusion in the presence of CH5183284 (A.ii), Erlotinib (B.ii), or SB431542 (C.ii). White asterisks denote HPEKp in the seam between adjacent spheroids. D: Relative cellular ATP content of HPEKp/HWJSC spheroids after 4 days of fusion as quantified using CellTiter Glo 3D assay following the manufacturer’s instructions, normalized to the DMSO control and presented as mean ± SEM for at least 12 replicates aggregated from 2 at least independent experiments. E: Quantification of the relative extent of HPEKp in the seam between spheroids, normalized to the day 0 time point, upon treatment with either vehicle control (white) or human recombinant EGF at 2 ng/mL (green), 10 ng/mL (blue), or 50 ng/mL (black) and presented as mean ± SEM for 14 replicates aggregated from 4 independent experiments. Asterisk denotes statistical significance relative to control at each time point with two-way ANOVA and Dunnett post-hoc test (α = 0.05). F: Area under the curve analysis of EGF-treated spheroids throughout fusion, tabulated using the trapezoid method, presented as mean ± SEM for 14 replicates aggregated from 4 independent experiments. The vehicle control (0 ng/mL EGF) is displayed as 0.1 ng/mL in the figure. Asterisk denotes statistical significance relative to control with one-way ANOVA and Fisher’s least significant difference test (α = 0.05).

We further interrogated the sensitivity of the *in vitro* spheroid fusion assay by perturbing pathways known to regulate palate fusion. We tracked the progression of fusion by quantifying the relative abundance of epithelial cells in the seams between fusing spheroids, consistent with a published method of quantifying palate fusion by mesenchymal confluency[27]. Chemical challenge with CH5183284 (FGFR inhibitor) or Erlotinib (EGFR inhibitor) significantly increased the relative extent of HPEKp in the seam between spheroids relative to the DMSO control. In particular, significantly more HPEKp were present in the seam between HWJSC spheroids at day 1 and day 4 of fusion upon treatment with CH5183284 (Fig 7A) and at day 2 and 4 upon treatment with Erlotinib (Fig 7B) relative to the DMSO control. An inhibitor against transforming growth factor β receptor signaling (SB431542) elicited no significant effect on HPEKp in the seam at any time points relative to the DMSO control (Fig 7C). Interestingly, although we observed more HPEKp in the seams between fusing spheroids upon CH5183284 treatment, we observed no influence of CH5183284 on the total equivalent diameter of fusing HWJSC/HPEKp spheroids (S5 Fig). This observation suggests that FGF inhibition did not influence the fusion of the mesenchymal spheroids but reduced the ability of HPEKp to be removed from the seams between spheroids. This highlights an important aspect of the *in vitro* fusion model, which is that quantitation of either fusing tissue size or HPEKp presence in the spheroid seams informs independent aspects of complete fusion that are also required for fusion of the palatal shelves *in vivo*. Future studies are necessary to confirm the specific role of EGF and FGF signaling in spheroid fusion *in vitro*, as sustained inhibition of either pathway over 4 days in culture resulted in a significant reduction in cellular ATP content relative to the DMSO control (Fig 7D). Finally, stimulation of the *in vitro* fusion model with 10 ng/mL of human recombinant EGF significantly increased the extent of HPEKp in the seam between spheroids at day 4 relative to the vehicle control, whereas stimulation by either 2 ng/mL or 50 ng/mL elicited no significant effect (Fig 7E). Analysis of the area under the curve in each treatment group revealed a significantly higher area under the curve (time x extent of HPEKp in the seam) only in the 10 ng/mL EGF condition relative to the vehicle control (Fig 7F). These data suggest that in the spheroid fusion model, FGF signaling and a balance of EGF signaling are both required for *in vitro* fusion in agreement with the role of FGF and EGF signaling during secondary palate fusion *in vivo*.

## Discussion

This study is the first report of a three-dimensional approach to engineer epithelial-stromal interactions and to recapitulate palatal architecture, phenotype, and fusion *in vitro* using human cells. Here, we generated osteogenic HWJSC spheroids and coated the outer spheroid surface with epithelial progenitor cells. HWJSC spheroids after 7 days of culture in osteo-induction medium expressed prototypical basement membrane proteins (collagen I, collagen IV, laminin, fibronectin) that constitute the likely mechanism whereby epithelial cells attached to the outer spheroid surface. We observed that fusion of engineered HWJSC/HPEKp spheroids captured a key feature of palatal fusion—removal of the epithelial cells from the seams between spheroids—that was dependent on EGF and FGF signaling. We did not observe any evidence that CellTracker Green stained HPEKp cells, or their remnants, remained in the seam between fusing HWJSC/HPEKp spheroids, which could suggest that the mechanism of fusion was likely migration of epithelial cells out of the seam and likely not EMT or apoptosis, though further studies are warranted to examine this hypothesis. Tissue fusion and organization during embryonic development has been hypothesized to involve tissue biophysics and differential cell-cell and cell-matrix adhesion according to the differential adhesion hypothesis[2]. Many previous studies have examined the assembly and fusion of spheroids containing adhesion-dependent cell types[28–30], and spheroid fusion was shown to involve tissue surface tension[29] and cytoskeletal dynamics[31]. Indeed, literature demonstrates that cytoskeletal dynamics are critical for complete palatal shelf fusion *ex vivo*[32]. The fusion of heterotypic spheroids here (S5 Fig) was likely driven by biophysical forces and cytoskeletal dynamics as suggested by these prior studies, while the removal of epithelial cells from the seams between spheroids (Fig 6) was lively driven by cytoskeletal dynamics and differential cell-cell and cell-matrix adhesion. Cell sorting during spheroid assembly has been demonstrated to varying degrees with different epithelial and stromal cell lines[33,34]. However, in our preliminary studies (data not shown), spheroids composed of intermixed epithelial and mesenchymal cells failed to properly ‘sort out’ to organoids with epithelial cells restricted to the outer mesenchymal spheroid surface, which agrees with a similar inability of outer root sheath keratinocytes and dermal papilla cells to self-organize when co-seeded into spheroids[35]. This observation suggests that cell sorting out can occur during fusion of HWJSC/HPEKp spheroids, but not during generation of HWJSC/HPEKp spheroids themselves, which warrants future investigation into the biophysical and molecular characteristics of tissue fusion and how spheroid fusion is distinct from spheroid assembly. Many prior studies have shown that spheroid fusion can promote macroscale tissue formation *in vitro* for regenerative medicine applications[36,37], and the co-cultured spheroid fusion model here is sensitive to chemical disruption and is amenable to developmental toxicology applications.

Complete fusion between HWJSC/HPEKp spheroids here, as defined by the coalescence of mesenchymal spheroids and removal of the epithelial cells from the seams between spheroids, was dependent on signaling from EGF and FGF. Inhibition or agonism of EGF signaling resulted in HPEKp persistence in the seam between spheroids, which provides evidence of the failure of co-cultured spheroids to completely fuse upon perturbation of EGF signaling. Given that HPEKp expressed higher levels of *EGF* and *EGFR* relative to day 7 HWJSC spheroids (S6 Fig), these data suggest that disruption of EGF signaling likely perturbed EGF-dependent epithelial cell morphogenesis. The disruption of *in vitro* spheroid fusion upon treatment with 10 ng/mL EGF but not 2 ng/mL or 50 ng/mL is consistent with the non-monotonic dependence of epithelial cell function on EGF stimulation, wherein over-stimulation of the EGF pathway has been shown to reduce responsiveness of epithelial cells to exogenous EGF[38]. The dependence of spheroid fusion on a balance of EGF signaling is supported by evidence that explanted mouse palates fail to completely fuse (as evidenced by the presence of MEE cells in the seam) upon genetic ablation of *Egfr*[39] and by evidence of a failure of explanted palatal shelves to fuse upon stimulation by exogenous EGF[40]. Our data are also supported by evidence of a balance of EGF and TGFβ signaling predicted to be important for palate fusion by an *in silico* agent-based model[12], though future work is warranted to explain why disruption of TGFβ signaling had no effect in the *in vitro* fusion model. We also observed that inhibition of FGF signaling resulted in HPEKp persistence in the seam between spheroids, suggesting a failure of complete fusion. FGF signaling is known to play a role in palate fusion, as *Fgf10* and *Fgfr2b* knockout mice exhibit cleft palate, likely due to reduced mesenchymal proliferation and associated failure of palatal elevation and outgrowth. However, *ex vivo* explanted palates from *Fgfr2b* knockout mice still exhibit fusion *in vitro*, suggesting that FGFR2 signaling may be necessary for palatal elevation but not necessarily fusion[41]. Literature also demonstrates that *Fgfr1* knockout mice exhibit cleft palate, and explanted palates from *Fgfr1* knockout mice exhibit a failure to fuse *in vitro* due to the presence of MEE cells in the seam[42], which suggests that while FGFR1 and FGFR2 signaling are both necessary for palatal elevation, FGFR1 signaling is necessary for removal of the MEE cells during palate fusion. CH5183284 inhibits the kinase activity of FGFR1, FGFR2, FGFR3, and to a lesser degree, FGFR4 [43]. Thus we posit that in the spheroid fusion model, inhibition of both FGFR2 activity (*FGFR2* was expressed primarily by HPEKp, S6 Fig) and FGFR1 activity (*FGFR1* was expressed in the HWJSC day 7 spheroids by RNASeq, S5 Table) blocked complete spheroid fusion due to effects in both the mesenchyme and the epithelium. We did not observe an influence of TGFβ pathway inhibition on HWJSC/HPEKp spheroid fusion, suggesting that spheroid fusion was not dependent on TGFβ signaling. We observed that HPEKp expressed higher levels of *TGFB3* than day 7 HWJSC spheroids, in agreement with the primarily epithelial expression pattern of *TGFB3* in palatal shelves prior to fusion[44]. Therefore, lack of TGFβ dependence in the spheroid fusion model is likely not due to lack of expression of TGFβ signaling components. The insensitivity of the spheroid fusion model to SB431542 here disagrees with the inhibitory effect reported for SB431542 on mouse palatal explant fusion *in vitro*, where the palatal shelves failed to completely fuse[27]. The data here suggest that the spheroid fusion model recapitulates EGF and FGF-dependent mechanisms of fusion *in vitro* for predictive toxicology applications, though future studies are needed to unravel the full complement of signaling pathways that initiate or drive fusion *in vitro*.

Transcriptomic and proteomic analyses of HWJSC spheroids before and after culture in OM suggest that HWJSC spheroids after 7 days exhibited an osteoblastic and pre-mineralization phenotype. Embryonic palatal shelves begin to express elevated levels of osteogenic markers prior to fusion, and mineralization (as observed *via* alizarin red staining) occurs several days after secondary palate fusion[13,21,45]. We observed an increase in osteogenic differentiation and skeletal system development genes *BGLAP* and *RUNX2* in HWJSC spheroids by day 7 in culture, though differentiation did not elicit changes in gene expression of osteogenesis-related genes *ALPL* or *SPP1* (*via* PCR or RNAseq) or *SP7* (*via* RNASeq, S5 Table). Upregulation of *MYC* is consistent with evidence of its role as a transcriptional regulator of osteogenic differentiation of mesenchymal stem cells[46], though early *MYC* expression has also been observed in human adipose stem cells undergoing adipogenic differentiation[47]. Whole-transcriptome analysis of day 7 HWJSC spheroids relative to day 1 HWJSC spheroids revealed an increase in genes associated with skeletal system development, organismal development, cell proliferation, and cell movement, and upregulated *RUNX2* and *FOXO1* expression during osteogenic differentiation (S5 Table) corroborates their role during mesenchymal stem cell osteogenic differentiation[48]. Future work is needed to explain both the increase (Fig 2B) and decrease (Figure C in S3 Fig) in genes related to morphogenesis and proliferation that we observed in day 7 versus day 1 HWJSC spheroids *via* RNASeq. We compared the up-regulated gene list from RNASeq (S5 Table) to a published data set examining each stage of mesenchymal stem cell osteogenic differentiation and observed that our data aligned with intermediate and late mesenchymal stem cell osteogenic differentiation prior to the mineralization stage. We observed increased expression of 4 out of 7 (*FBN1*, *IGF2*, *COL14A1*, *RUNX2*) and 6 out of 18 of the genes (*COMP*, *POSTN*, *SPARC*, *COL5A1*, *COL8A1*, *THBS1*) identified as intermediate or intermediate/late osteogenic differentiation genes, respectively, and in those same categories, we observed decreased expression in none of the 7 intermediate genes and 4 of the 18 (*ICAM1*, *PTK2*, *FZD6*, *THY1*) late osteogenic differentiation genes. In contrast, we observed increased expression of 2/14, 1/4, 1/5, and 3/8 and decreased expression of 6/14, 3/4, 2/5, and 3/8 of the genes identified as late osteogenic differentiation, early mineralization, intermediate mineralization, and late mineralization-associated genes, respectively[49]. Granchi *et al*. noted that genes associated with angiogenesis were increased throughout osteogenic differentiation[49], which agrees with the enrichment of cardiovascular development genes we observed in the RNASeq comparison of day 7 versus day 1 spheroids (Fig 2B and 2C). Our results corroborate findings of osteogenesis-angiogenesis coupling *in vivo*[50] that may have been mediated by the elevated expression of *CTGF* [51,52] and *HIF1A* [53] in HWJSC spheroids during osteogenic differentiation. We examined the top 10 upregulated genes in day 7 versus day 1 HWJSC spheroids and observed that 5 out of the 10 are directly (*ZBTB16* [54–56]) or indirectly related to osteogenesis or mineralization (*SAA1* and *SAA2* [57], *SLPI* [58], *IL1RL1* [59]), while upregulated *HMCN2* may be attributed to its role in calcium regulation and cell binding *via* gene ontology. We observed an increase in *STAT3* and *IL1RL1* expression in day 7 versus day 1 HWJSC spheroids consistent with elevated *IL1RL1* in a STAT3 overexpression model exhibiting osteoblast differentiation[60]. Two negative regulators of Wnt signaling, *APCDD1* and *TRABD2B*, were upregulated during HWJSC spheroid osteogenic differentiation, which is supported by evidence of elevated *APCDD1* expression during osteogenic differentiation[56,61] and corroborates the role of Wnt as a negative regulator of osteogenic differentiation. Increased *APOE* expression in day 7 HWJSC spheroids agrees with the observation that human mesenchymal stem cells produce APOE upon treatment with dexamethasone or osteo-induction medium[62]. Finally, we observed elevated expression of *SLC16A10*, encoding an aromatic amino acid transporter, in day 7 spheroids, which may highlight a previously undiscovered role of SLC16A10 during osteogenic differentiation.

While HWJSC spheroids primarily underwent osteogenic differentiation, we cannot rule out some adipogenic or chondrogenic differentiation. We compared our results to a transcriptomics analysis of HWJSC differentiation to adipose, cartilage, and bone lineages[63] and observed that the upregulated genes in day 7 versus day 1 HWJSC spheroids overlapped with 33%, 15%, and 14% of the genes specifically associated with osteogenic, adipocyte, and chondrocyte differentiation. During osteogenic differentiation, we observed a substantial increase in *PPARG* expression, a marker for adipogenesis, that raised the possibility that HWJSC spheroids underwent adipogenic differentiation during culture in OM. Adipogenesis occurs through transcriptional activation of CCAAT/enhancer binding protein β (C/EBPβ) followed by transcriptional activation of PPARγ and C/EBPα that, upon PPARγ ligand agonism, activate adipose-specific gene expression[64]. PPARγ is required during adipogenesis *in vitro*, and cells lacking C/EBPα require forced overexpression of PPARγ to restore adipogenic potential[65]. We did not observe any *CEBPA* transcripts or an increase in *CEBPB* expression during HWJSC spheroid osteogenic differentiation *via* RNASeq, and given that we did not observe induction of downstream targets of *PPARG* expression (*e*.*g*. *FABP4*, *ACOX1*) or inducers of endogenous *PPARG* ligand expression (*SREBF1*), we conclude the *PPARG* gene induction during osteogenic differentiation here was not correlated with adipogenic differentiation. We observed a greater than 10-fold increase in *FOXO1* expression during HWJSC spheroid osteogenic differentiation (S5 Table), which together with evidence that FOXO1 represses PPARγ target genes[66] suggests that elevated *PPARG* expression during osteogenic differentiation was disconnected with adipogenesis during osteo-induction of HWJSC spheroids. RNASeq performed on the day 7 HWJSC spheroids identified up-regulated genes associated with the GO terms ‘response to organic substance’ and ‘response to hormone’ (Fig 2B), both of which contain the gene *PPARG*, which may indicate the presence of a *PPARG*-inducing hormone in the OM. Dexamethasone is a common culture supplement used to promote osteogenic differentiation of mesenchymal stem cells[67,68], and literature supports that dexamethasone treatment of mesenchymal stem cells[69] or addition of dexamethasone to osteo-induction medium[70,71] upregulates *PPARG* expression *in vitro*. We performed LC-MS on the OM (S6 and S7 Tables) and confirmed the presence of a compound with identical product ions as dexamethasone (S7 Fig) and with an abundance of ~65 nM, indicating that *PPARG* expression here could have been driven by dexamethasone in the OM. In the presumed absence of a PPARγ agonist or increase in the expression of key PPARγ target genes, we conclude that HWJSC spheroids were either not undergoing or weakly undergoing adipogenic differentiation and primarily differentiated down the osteogenic lineage. Future work is necessary to confirm the relative contribution of osteogenesis, adipogenesis, and chondrogenesis in the HWJSC spheroid culture model, although our data altogether suggest that differentiation of HWJSC spheroids was primarily towards an osteogenic lineage. The data confirm that HWJSC spheroids exhibit phenotypic characteristics of palatal mesenchyme and basement membrane expression that support their use in modeling palatogenesis.

HWJSC spheroids exhibited changes in protein expression at day 7 that suggest a glycolytic metabolic shift during osteogenic differentiation. HWJSCs cultured in 2D have been shown to undergo osteogenic differentiation with a concomitant increase in dependence on oxidative phosphorylation for energy with no significant change in glycolysis[72]. Our results suggest that HWJSC spheroids upon osteogenic differentiation in 3D spheroid culture (as opposed to the observations from 2D culture) exhibited significantly increased protein expression associated with glycolysis and reactive oxygen scavenging (S3 and S4 Tables). The increase in *HIF1A* gene expression that we observed in day 7 HWJSC spheroids (S5 Table) suggests a mild hypoxic environment in day 7 HWJSC spheroids. This hypoxia was likely related to reactive oxygen species (ROS) generation[73], which may explain the observed increase in protein expression of reactive oxygen detoxifying proteins superoxide dismutase-2 (SODM) and peroxiredoxin-6 (PRDX6) at day 7 of differentiation versus day 1. Increased expression of *HIF1A* and ROS detoxifying proteins suggest that HWJSC osteogenic differentiation may have been associated with mild hypoxia, generation of ROS, and increased dependence on glycolysis. Oxidative phosphorylation during osteogenic differentiation in 2D contexts may thus indicate high oxygen availability during differentiation, while our results suggest that the metabolic state associated with osteogenesis is highly dependent on the context of differentiation. Finally, we compared the ontology of upregulated proteins at day 7 and observed that 1 out of 22 of the upregulated proteins were associated with osteoblast differentiation whereas none was associated with fat cell or chondrocyte differentiation consistent with our conclusion that HWJSC spheroid differentiation was primarily down the osteogenic lineage. NAMPT was upregulated in day 7 versus day 1 spheroids, and recent evidence of NAMPT-dependent osteogenic differentiation of mesenchymal stem cells[24] suggests that elevated NAMPT protein expression here may be associated with HWJSC spheroid osteogenic differentiation. Four of the upregulated proteins in day 7 HWJSC spheroids (HSPB1, ANXA6, NNMT, and SODM) and one down-regulated protein (VIM) were also observed in a similar proteomics assessment of chondrogenic differentiation of HWJSC aggregates at day 7 of differentiation[74], which, given the lack of chondrogenesis-related proteins in our 2D-DIGE analysis, suggests that the differential expression of these proteins is associated with 3D culture of HWJSCs.

## Conclusion

We describe a method to engineer palate-like organoids, using human cells, with similar architecture and osteogenic phenotype of embryonic palate. Multipotent stem cell spheroids mimic the mesenchymal compartment of embryonic palate, and through culture in osteo-induction medium, we identified a duration and condition of culture that elicited gene and protein expression changes consistent with osteoblast differentiation. We show that the primarily osteogenic mesenchymal spheroids supported the attachment of epidermal progenitor cells on their outside spheroid surface, which may be influenced by expression of matrix proteins produced during differentiation in spheroid culture. An acceptable level of epithelial coverage on mesenchymal spheroids, important for mimicking the epithelial layer of embryonic palates, was dependent on the seeding density of epithelial cells and the physical size of the mesenchymal spheroids. We developed a quantitative approach for evaluating fusion between spheroids based on assessing the absence of epithelial cells between contacting spheroids. Finally, an important feature of the spheroid fusion model was the demonstration of EGF- and FGF-dependent fusion behavior, consistent with studies of palate fusion. The model system developed here will be useful for prioritizing chemicals that are likely to perturb palate fusion and more broadly may serve as a novel method to study epithelial-stromal interactions and *in vitro* fusion behavior in other developmental processes for toxicity assessment.

## Supporting information

S1 Fig Representative brightfield microscopy images of HWJSC spheroids over time. Images were captured using Olympus CK40 light microscope equipped with 4X objective and a color RS Photometrics camera. Images were acquired and analyzed to determine the mean diameter using NIS Elements v4.3 software (Nikon). Representative images from each condition at day 1, 7, 14, and 21 were cropped uniformly and converted to black and white. Scale bar represents 200 μm.
(TIF)

S2 Fig qRT-PCR data for housekeeping gene, pluripotency genes, and osteogenesis-related genes. A-H: qRT-PCR fold change data (presented on a log2 axis) over time in culture of HWJSCs spheroids. All data were normalized to the GAPDH housekeeping gene and the day 1 time point, and statistical comparisons were made relative to a mean value of ‘1’ using a two-tailed t-test at α = 0.05. No statistical differences were observed across any of the 8 probes measuring *ACTB* (A), *NANOG* (B), *SOX2* (C), *KLF4* (D), *POU5F1* (E), *ENG* (F), *ALPL* (G), or *SPP1* (H).
(TIF)

S3 Fig Quality control analysis of transcriptomics data and down-regulated gene ontology. A: 3D plot of principal component analysis of RPKM values from day 1 and day 7 HWJSC spheroid samples from three biological replicates. B: Plot of fold change identified from RNAseq (y-axis) and qRT-PCR (abscissa). Black dots represent data points that were statistically significant using both RNASeq and PCR, blue dots represent data points that were statistically significant on RNASeq and not PCR, red dots represent data points that were statistically significant on PCR and not RNASeq, and open circles represent data points that were not statistically significant on either RNASeq or PCR. Not shown is BGLAP, which although it was statistically up-regulated via PCR (fold change 3.35) was not identified on RNASeq. C: GO analysis using v6.8 DAVID functional annotation clustering of down-regulated genes comparing day 7 to day 1 HWJSC spheroids. A representative GO term (with the highest number of genes represented and a Bonferroni p-value < 0.05) was chosen from the top 10 enriched clusters.
(TIF)

S4 Fig Correlation between proteomics fold change and RNASeq fold change (day 7 versus day 1). A: Cluster plot of proteomics fold change (ANOVA *p*-value<0.055) graphed against RNASeq fold change (FDR step-up < 0.05), with labels indicating the name of each gene. The Pearson correlation coefficient (r) for the 12 compared data points was 0.8560. B: Cluster plot of proteomics fold change (ANOVA *p*-value<0.055) graphed against RNASeq fold change, with filled spots indicating RNASeq FDR step-up < 0.05 and open circles indicating no statistical significance in the RNASeq data set. The correlation between protein-level and gene-level changes with no significance threshold is less strong than with the threshold (Pearson correlation coefficient 0.5285).
(TIF)

S5 Fig Equivalent tissue diameter of fusing HWJSC/HPEKp spheroids over time. Fusing HWJSC/HPEKp spheroids were imaged using confocal microscopy on days 0, 1, 2, and 4, and the red channel (representing HWJSCs) was isolated for each z-slice. Background fluorescence was eliminated from each z-slice, and a maximum intensity projection was generated for each sample z-stack. The mean equivalent diameter was calculated from three aggregated replicates (one replicate from three independent experiments) and normalized to the day 0 time point. Samples were treated either with DMSO or with 20 μM of CH5183284. Two-way ANOVA demonstrated a significant contribution of fusion time (*p*-value < 0.05) on the normalized equivalent diameter. There was no difference in the equivalent diameter of the fusing spheroids between the CH5183284-treated samples relative to the DMSO control at any time point.
(TIF)

S6 Fig Gene expression analysis of HWJSC spheroids versus HPEKp alone via qRT-PCR. Fold change in gene expression comparing HWJSC spheroids to HPEKp cultured alone on microcarrier beads. Data for each gene probe (shown on the y-axis) are presented as the mean ± SD fold change in expression for each gene of HWJSC spheroids relative to HPEKp, except the error bars for KRT17 were omitted because they extend beyond the axis limit. Results demonstrated that ENG, VIM, and AHR were expressed more highly in HWJSCs than HPEKp, and EGFR, TGFB3, EGF, FGFR2, ITGA6, and KRT17 were expressed more highly in HPEKp than HWJSCs. These data corroborate ENG and VIM as cell-specific gene markers for mesenchymal cells and ITGA6 and KRT17 as cell-specific gene markers for epithelial cells.
(TIF)

S7 Fig LC-MS spectra for OM in comparison with dexamethasone standard. A: Product ion spectrum of dexamethasone (m/z 393.2); B: Product ion spectrum of the ion of m/z 393.2 from an aliquot of OM diluted with acetonitrile.
(TIF)

S1 Table List of all primers used in qRT-PCR experiments. The table contains a list of all primers used in qRT-PCR experiments (data in Fig 1, S2, S3 and S6 Figs) containing the gene symbol, gene name, Entrez gene ID, and TaqMan probe ID.
(XLSX)

S2 Table Full detailed list of upstream regulators from IPA analysis with target molecules. The table contains the full list of upstream regulators from IPA analysis of RNASeq data of day 7 versus day 1 HWJSC spheroids presented in Fig 2, including the upstream activator or inhibitor, z-score, p-value, and the target downstream molecules.
(XLSX)

S3 Table Full data from 2D-DIGE analysis of osteogenic differentiation at day 7 vs day 1. The table contains the full list of differentially expressed proteins in day 7 versus day 1 HWJSC spheroids (with 20% fold change, ANOVA *p*-value < 0.055). Presented are the protein name, protein symbol, Swiss-Prot accession number, SameSpots ID #, ANOVA *p*-value, fold change of protein expression at day 7 versus day 1, and the number of identified peptides from each of three gels representing biological replicates. Only the identified proteins with at least 2 identified peptides from at least 1 gel are presented in the table.
(XLSX)

S4 Table 2D-DIGE analysis with Ingenuity Pathway Analysis. The table contains a list of the top canonical pathways enriched for the differentially expressed proteins identified with 2D-DIGE in day 7 versus day 1 HWJSC spheroid samples (Fig 3). Included are the top canonical pathway, the p-value, and the % overlap from the IPA core analysis.
(XLSX)

S5 Table RNA sequencing data of osteogenic differentiation at day 7 vs day 1. The table contains a list of all of the genes identified from RNASeq analysis of the day 1 and day 7 HWJSC spheroids. Included in the table are the gene ID, the total number of reads, and differential expression analysis comparing the day 7 versus the day 1 samples (p-value, FDR step-up corrected p-value, ratio of expression of each gene of the day 7 versus day 1 samples, fold change of expression of each gene in the day 7 versus day 1 samples, and the LS mean).
(XLSX)

S6 Table Peak list from product ion spectra of dexamethasone and the ion of m/z 393.2 in cell media. Abundances are relative to the most abundant fragment ion, m/z 373.1. The table contains the ion masses and relative abundance of a dexamethasone standard (Cayman Chemical) versus the 393.2 m/z species identified from LC-MS analysis of the osteo-induction medium (OM).
(XLSX)

S7 Table MRM area ratios observed for dexamethasone and Betamethasone standards and the ion of m/z of 393.2 in OM. The ranges presented are calculated from the average multiple reaction monitoring (MRM) area ratios ± 3 standard deviations obtained from triplicate measurements of Dexamethasone and Betamethasone standards, and aliquots of OM diluted with acetonitrile.
(XLSX)

S1 File Supplemental methods. The file contains additional methodological details for the alkaline phosphatase activity assay with HWJSC spheroids, the gene expression analysis by qRT-PCR, the harvesting of HWJSC spheroids for 2D-DIGE, the immunofluorescence cell staining of day 1 and day 7 HWJSC spheroids, and the analytical determination of dexamethasone presence and abundance in the osteo-induction medium.
(DOCX)
