# Supplementary figures and images for: Engineering human cell spheroids to model embryonic tissue fusion in vitro
Source: PLoS One. 2017 Sep 12;12(9):e0184155. doi: 10.1371/journal.pone.0184155 (PMC5595299; doi:10.1371/journal.pone.0184155)

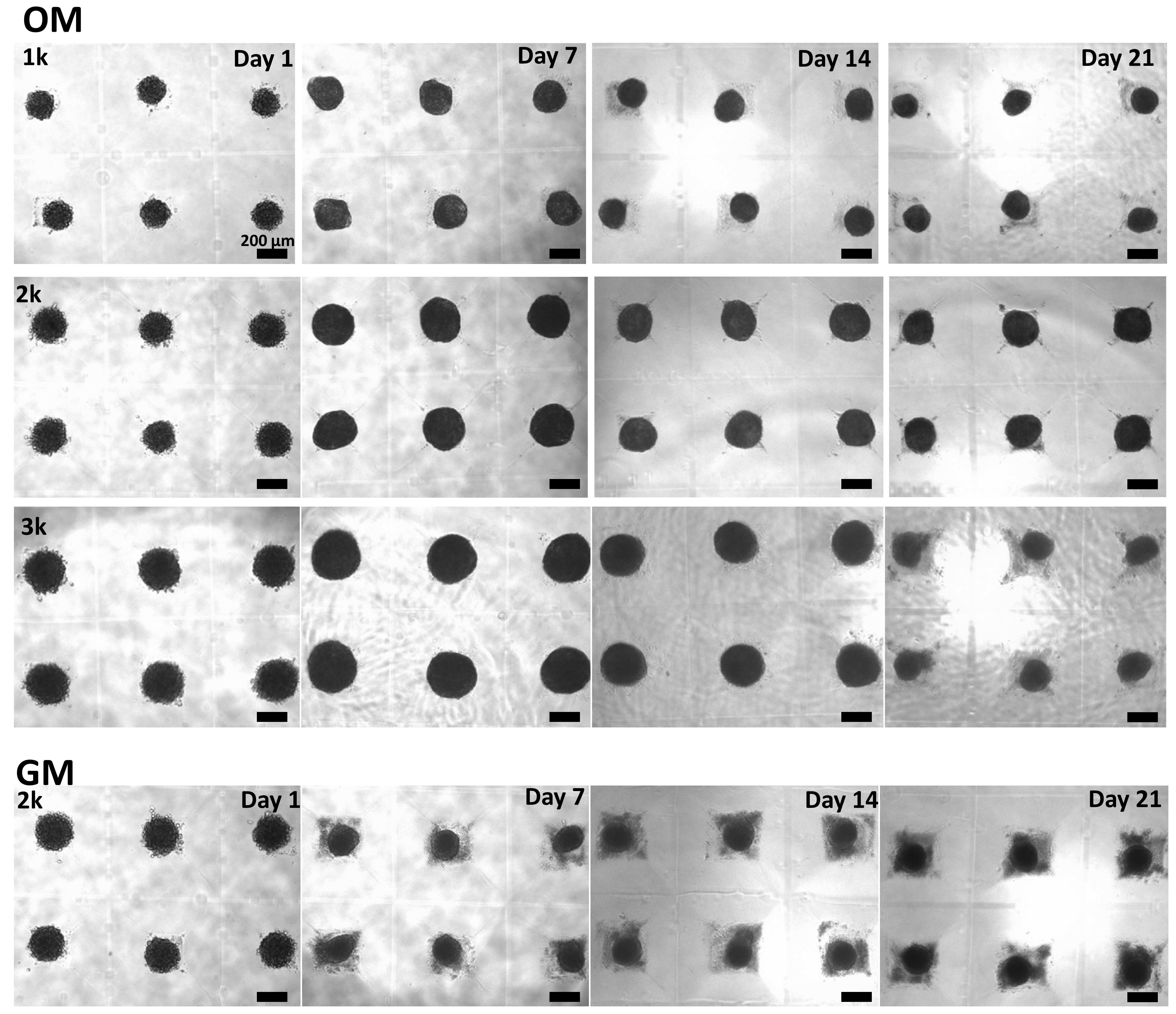

Supplement: S1 Fig — Images were captured using Olympus CK40 light microscope equipped with 4X objective and a color RS Photometrics camera. Images were acquired and analyzed to determine the mean diameter using NIS Elements v4.3 software (Nikon). Representative images from each condition at day 1, 7, 14, and 21 were cropped uniformly and converted to black and white. Scale bar represents 200 μm. (TIF) [file pone.0184155.s001.tif]

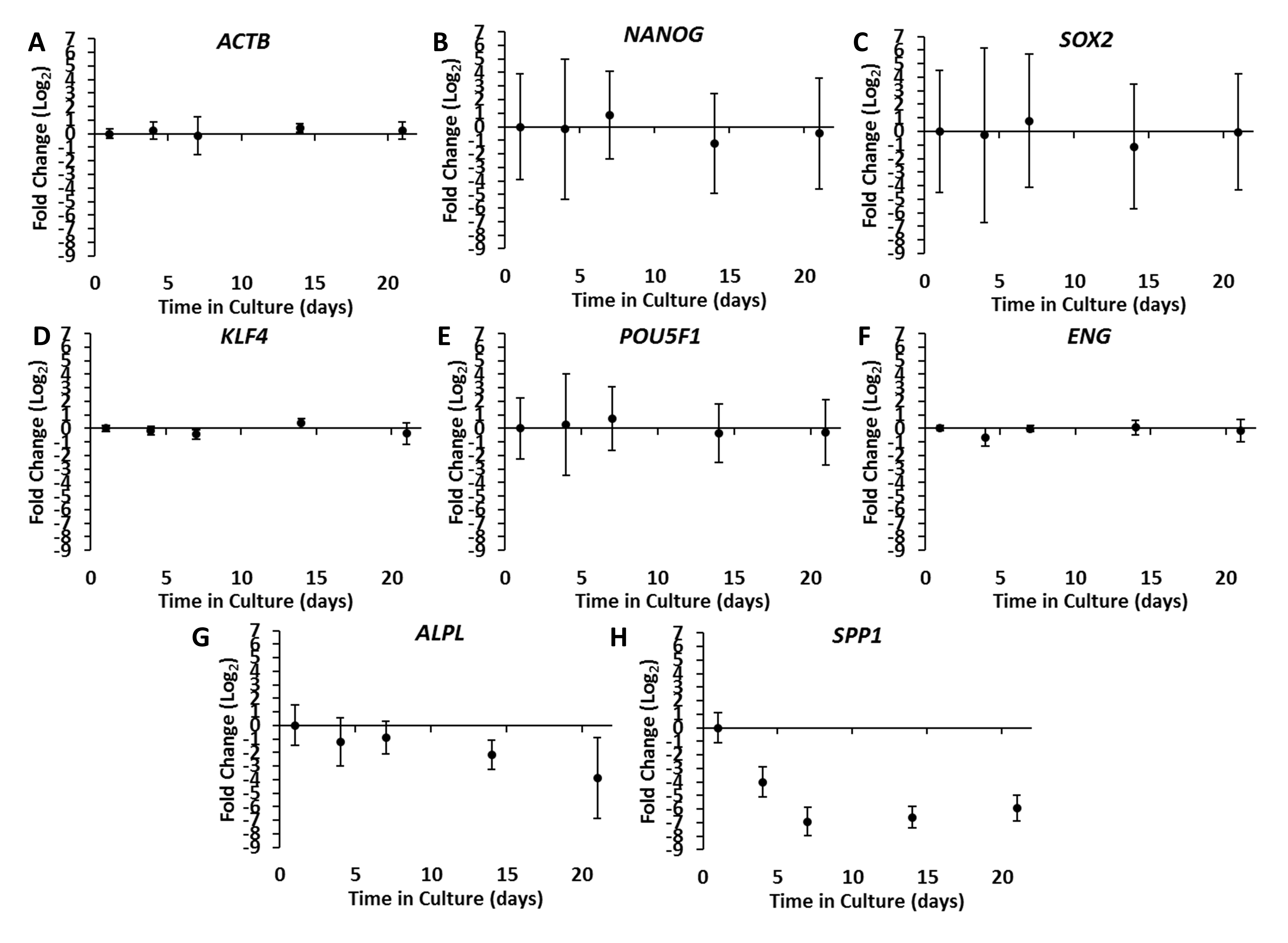

Supplement: S2 Fig — A-H: qRT-PCR fold change data (presented on a log2 axis) over time in culture of HWJSCs spheroids. All data were normalized to the GAPDH housekeeping gene and the day 1 time point, and statistical comparisons were made relative to a mean value of ‘1’ using a two-tailed t-test at α = 0.05. No statistical differences were observed across any of the 8 probes measuring ACTB (A), NANOG (B), SOX2 (C), KLF4 (D), POU5F1 (E), ENG (F), ALPL (G), or SPP1 (H). (TIF) [file pone.0184155.s002.tif]

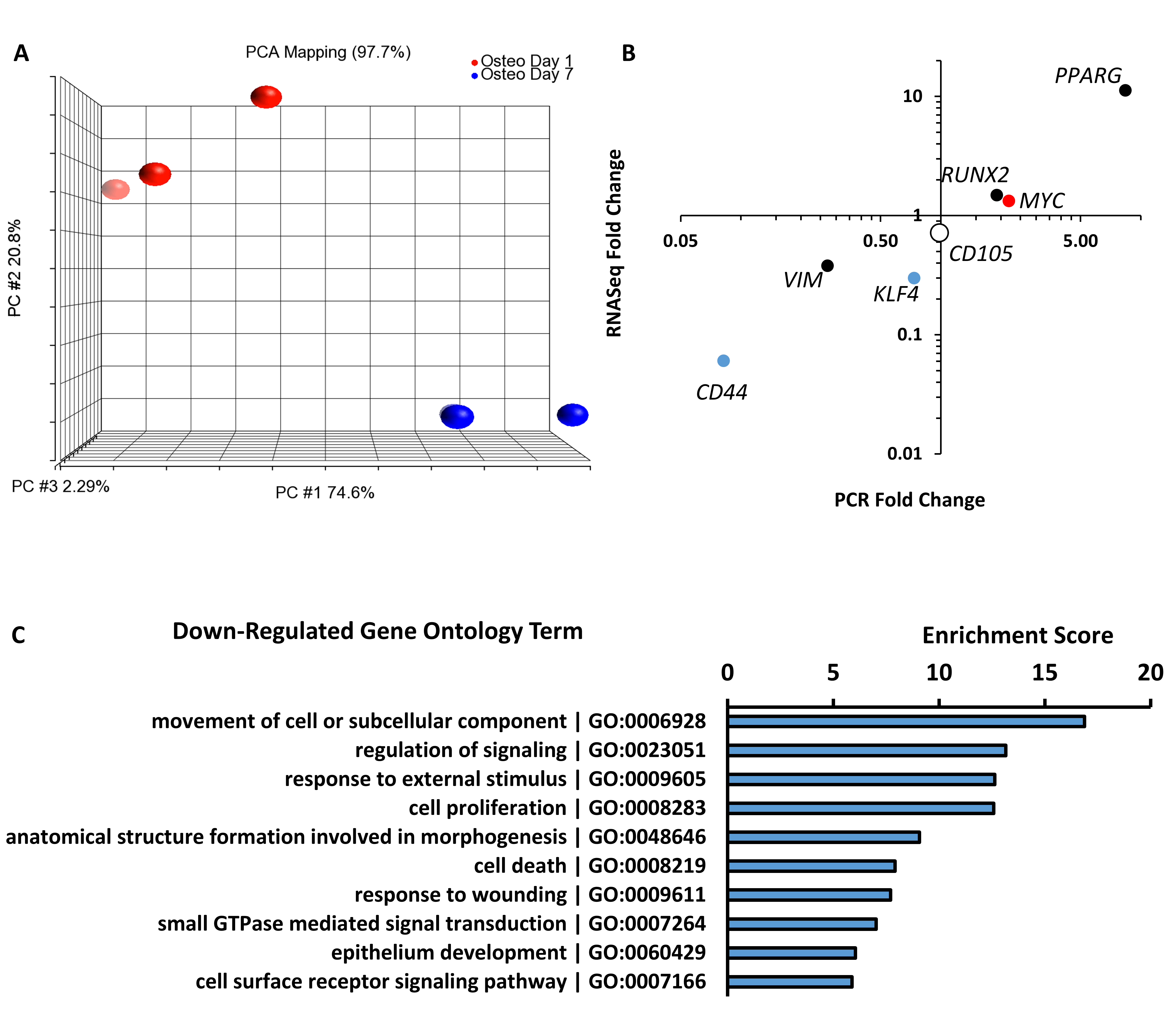

Supplement: S3 Fig — A: 3D plot of principal component analysis of RPKM values from day 1 and day 7 HWJSC spheroid samples from three biological replicates. B: Plot of fold change identified from RNAseq (y-axis) and qRT-PCR (abscissa). Black dots represent data points that were statistically significant using both RNASeq and PCR, blue dots represent data points that were statistically significant on RNASeq and not PCR, red dots represent data points that were statistically significant on PCR and not RNASeq, and open circles represent data points that were not statistically significant on either RNASeq or PCR. Not shown is BGLAP, which although it was statistically up-regulated via PCR (fold change 3.35) was not identified on RNASeq. C: GO analysis using v6.8 DAVID functional annotation clustering of down-regulated genes comparing day 7 to day 1 HWJSC spheroids. A representative GO term (with the highest number of genes represented and a Bonferroni p-value < 0.05) was chosen from the top 10 enriched clusters. (TIF) [file pone.0184155.s003.tif]

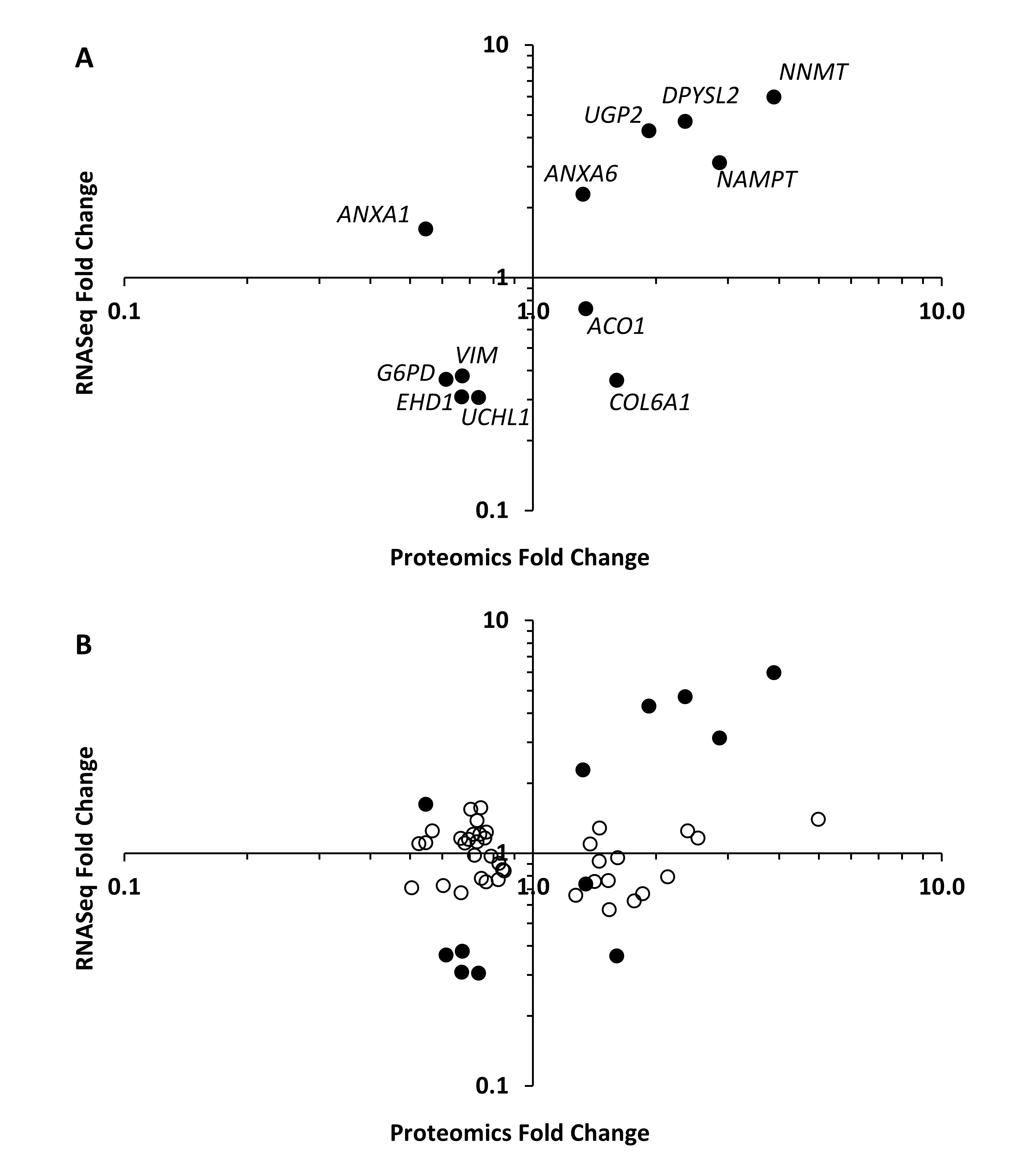

Supplement: S4 Fig — A: Cluster plot of proteomics fold change (ANOVA p-value<0.055) graphed against RNASeq fold change (FDR step-up < 0.05), with labels indicating the name of each gene. The Pearson correlation coefficient (r) for the 12 compared data points was 0.8560. B: Cluster plot of proteomics fold change (ANOVA p-value<0.055) graphed against RNASeq fold change, with filled spots indicating RNASeq FDR step-up < 0.05 and open circles indicating no statistical significance in the RNASeq data set. The correlation between protein-level and gene-level changes with no significance threshold is less strong than with the threshold (Pearson correlation coefficient 0.5285). (TIF) [file pone.0184155.s004.tif]

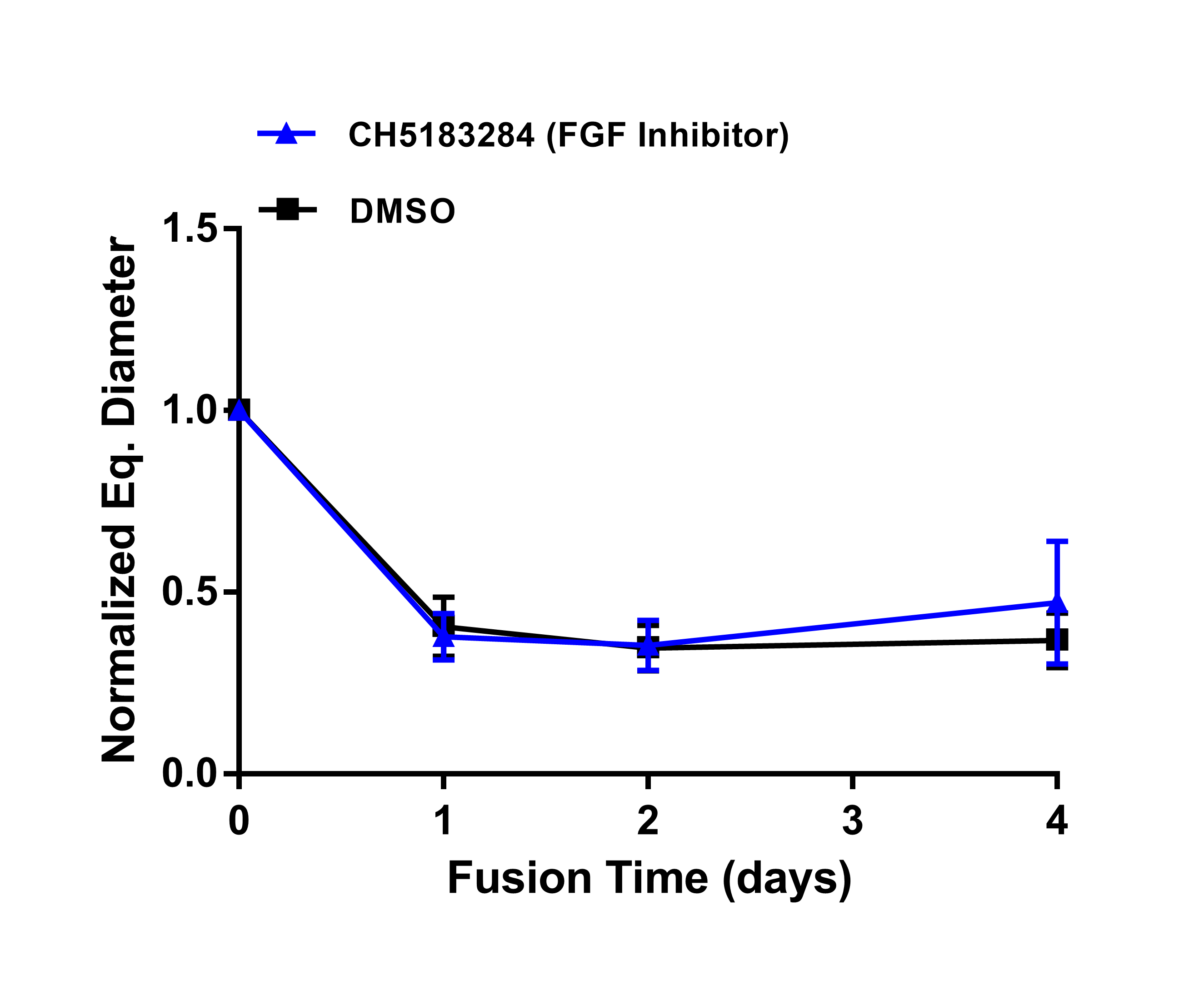

Supplement: S5 Fig — Fusing HWJSC/HPEKp spheroids were imaged using confocal microscopy on days 0, 1, 2, and 4, and the red channel (representing HWJSCs) was isolated for each z-slice. Background fluorescence was eliminated from each z-slice, and a maximum intensity projection was generated for each sample z-stack. The mean equivalent diameter was calculated from three aggregated replicates (one replicate from three independent experiments) and normalized to the day 0 time point. Samples were treated either with DMSO or with 20 μM of CH5183284. Two-way ANOVA demonstrated a significant contribution of fusion time (p-value < 0.05) on the normalized equivalent diameter. There was no difference in the equivalent diameter of the fusing spheroids between the CH5183284-treated samples relative to the DMSO control at any time point. (TIF) [file pone.0184155.s005.tif]

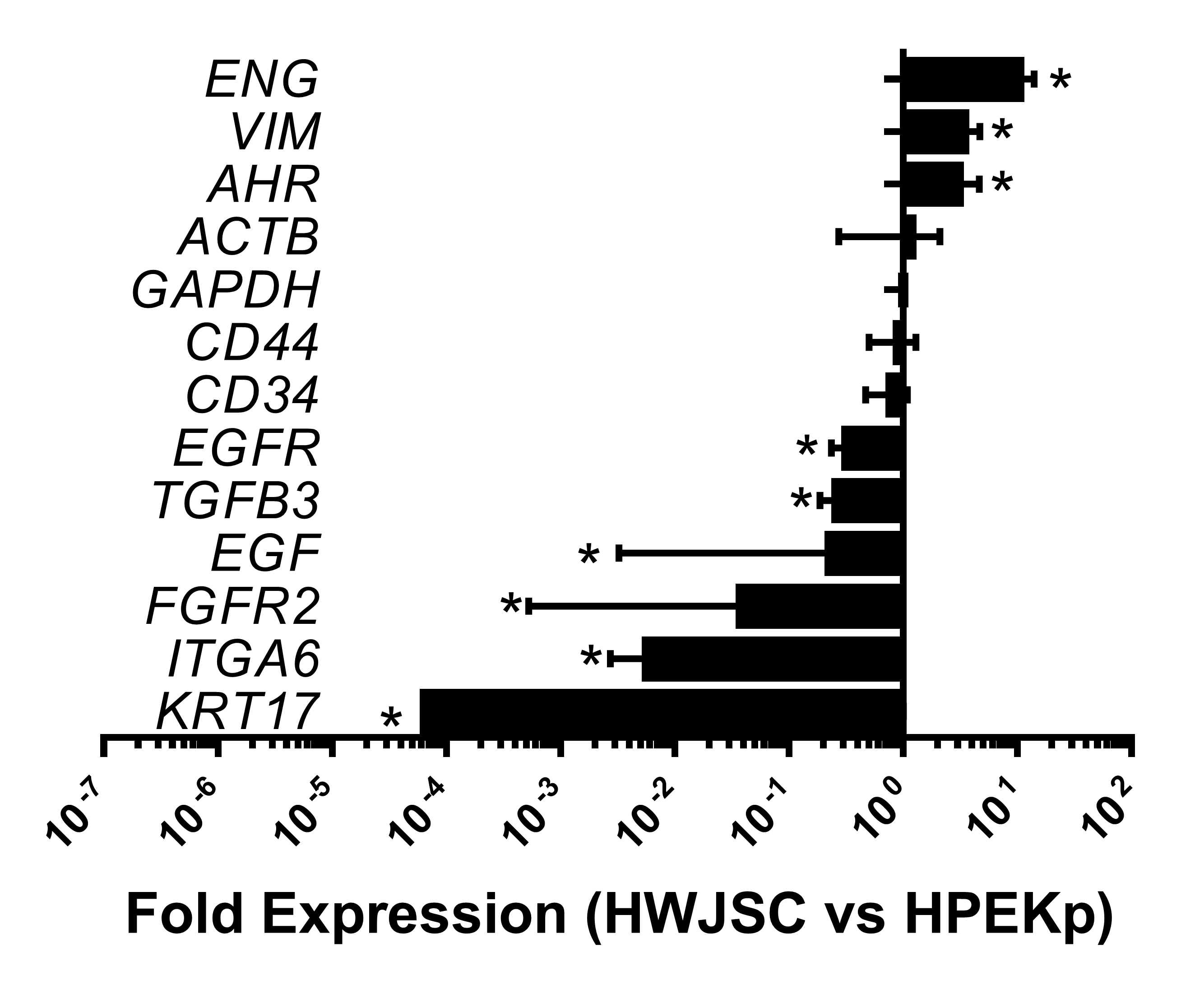

Supplement: S6 Fig — Fold change in gene expression comparing HWJSC spheroids to HPEKp cultured alone on microcarrier beads. Data for each gene probe (shown on the y-axis) are presented as the mean ± SD fold change in expression for each gene of HWJSC spheroids relative to HPEKp, except the error bars for KRT17 were omitted because they extend beyond the axis limit. Results demonstrated that ENG, VIM, and AHR were expressed more highly in HWJSCs than HPEKp, and EGFR, TGFB3, EGF, FGFR2, ITGA6, and KRT17 were expressed more highly in HPEKp than HWJSCs. These data corroborate ENG and VIM as cell-specific gene markers for mesenchymal cells and ITGA6 and KRT17 as cell-specific gene markers for epithelial cells. (TIF) [file pone.0184155.s006.tif]

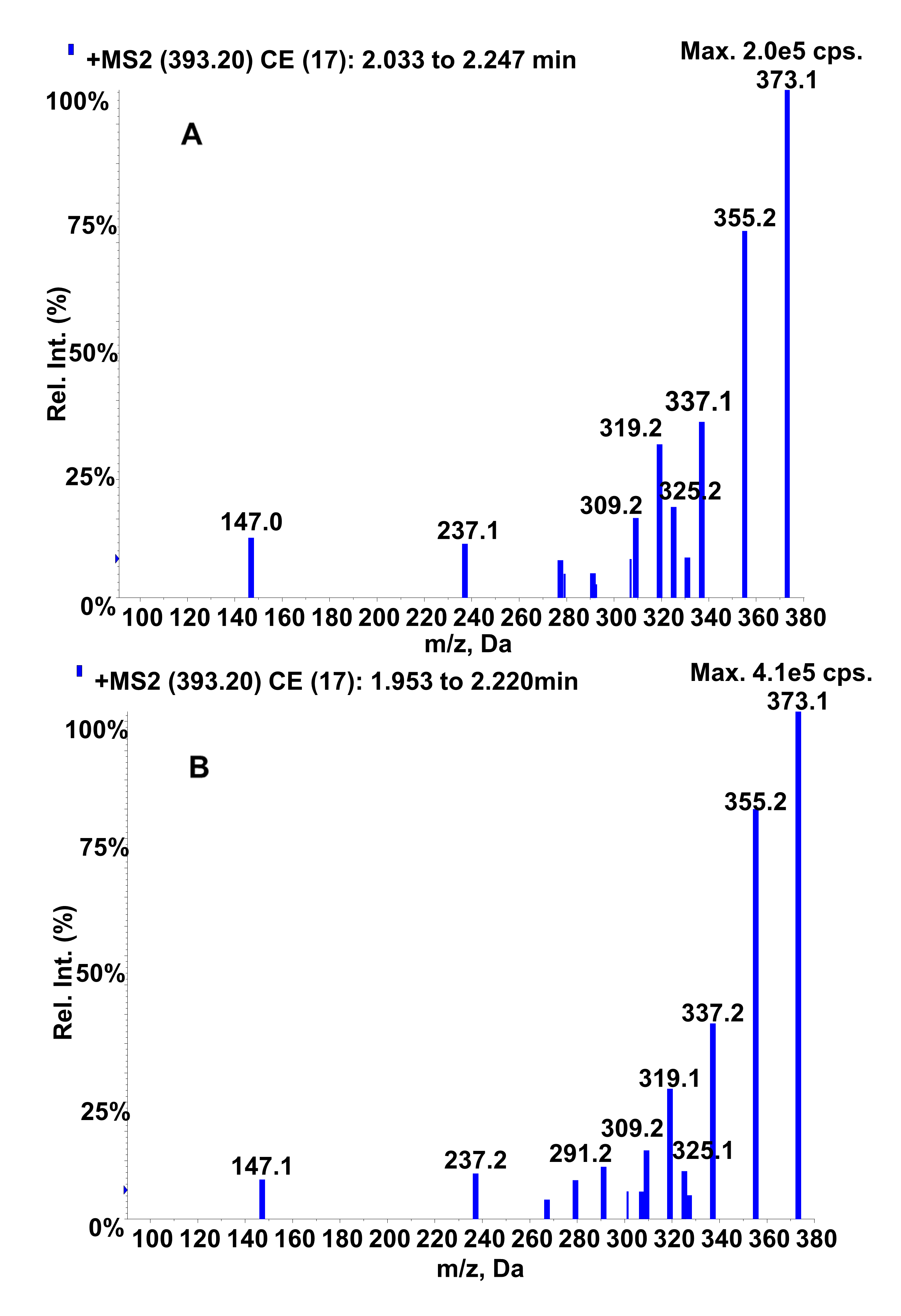

Supplement: S7 Fig — A: Product ion spectrum of dexamethasone (m/z 393.2); B: Product ion spectrum of the ion of m/z 393.2 from an aliquot of OM diluted with acetonitrile. (TIF) [file pone.0184155.s007.tif]
