# Supplementary material for: Engineering human cell spheroids to model embryonic tissue fusion in vitro
Source: PLoS One. 2017 Sep 12;12(9):e0184155. doi: 10.1371/journal.pone.0184155 (PMC5595299; doi:10.1371/journal.pone.0184155)
Supplement: S1 File — The file contains additional methodological details for the alkaline phosphatase activity assay with HWJSC spheroids, the gene expression analysis by qRT-PCR, the harvesting of HWJSC spheroids for 2D-DIGE, the immunofluorescence cell staining of day 1 and day 7 HWJSC spheroids, and the analytical determination of dexamethasone presence and abundance in the osteo-induction medium. (DOCX) [file pone.0184155.s015.docx]

Engineering human cell spheroids to model embryonic tissue fusion *in vitro*.

David G. Belair, Cynthia J. Wolf, Carmen Wood, Hongzu Ren, Rachel Grindstaff, William Padgett, Adam Swank, Denise MacMillan, Anna Fisher, Witold Winnik, Barbara D. Abbott

DOI: 10.1371/journal.pone.0184155

**Supplemental Methods**

**Alkaline Phosphatase Activity Assay**

HWJSC spheroids from a single well (300 spheroids) were harvested at each time point by removing medium, washing spheroids with DPBS, transferring to microcentrifuge tubes, and centrifuging at 2,000 x g for 10 s to remove supernatant. Spheroids were washed again in DPBS and subsequently incubated with alkaline phosphate substrate (prepared in DPBS according to the manufacturer’s instructions) for 4 hours at room temperature with rotation. Spheroids were then washed in DPBS and fixed in 4% formaldehyde (Sigma; prepared fresh in DPBS) and 1% Triton X-100 (Sigma) in DPBS for 3 hours at 4°C rotating. HWJSC spheroids were washed and stored at 4°C in DPBS. Stained HWJSC spheroids were transferred to optical 96 well plates (Thermo Scientific) and imaged using a Nikon A1 confocal laser scanning microscope equipped with Galvano scanner, dichroic mirror for 405/488/561/640 nm wavelengths, filters for 450/50, 525/50, and 595/50 nm wavelengths, lasers for 401.4, 488, 561.8, and 638 nm wavelengths, and 10X Plan Apo objective. HWJSC spheroids were imaged using z-stacks consisting of 300 µm range and 50 µm step size, and z-stacks were transformed into maximum intensity projections and analyzed to calculate spheroid fluorescence intensity using NIS Elements v3.2 software (Nikon). The mean fluorescence intensity calculated from at least 20 spheroids from three independent experiment was calculated and normalized to Day 1 spheroids. Statistical analysis was performed using two-way ANOVA and Tukey’s multiple comparison post-hoc test at a significance level of α=0.05.

**Gene Expression Analysis by qRT-PCR**

HWJSC spheroids from a single well were harvested at the designated times, transferred to separate blue Kontes microcentrifuge tubes (Kimble), washed in DPBS, and incubated in 10 µL of lysis buffer per sample tube for three freeze/thaw cycles on dry ice. Sample volume was brought up to 49.5 μL lysis buffer, and spheroids were homogenized for three cycles of 40 s each with Kontes Pellet Pestle (Kimble) followed by centrifugation at 12,000 x g for 5 min. Subsequently, 0.5 µL DNase was mixed with samples for 8 min at room temperature, followed by mixing with 5 µL of stop solution for 8-10 min at room temperature and storage at -20°C. Samples were reverse transcribed by mixing 18 µL lysate, 22.5 µL nuclease-free water (Qiagen), 4.5 µL RT enzyme (Thermo Cells-to-CT), and 45 µL RT buffer (Thermo Cells-to-CT) and incubating at 37°C for 1 hr, 95°C for 5 min, and 4°C until used in the PCR reaction. Qiagility pipettor (Qiagen) was used to mix 4 µL per well of reverse transcribed sample or water and 16 µL of solution containing primer (20X TaqMan probes; Thermo Fisher), master mix (2X TaqMan; Thermo Fisher), and water at a 1:5:10 volumetric ratio. PCR amplification was performed using an ABI 7900HT thermocycler, and C_T_ values were calculated using the ABI software using *GAPDH* as the reference gene based on its stability over time (± 2 C_T_). All samples were run in biological triplicate and normalized to the ‘Day 0’ time point using the 2^-ΔΔCT^ method. Statistical comparisons were made on normalized values relative to a mean value of ‘1’ using a two-tailed t-test at α=0.05.

For the comparison of HWJSC spheroid versus HPEKp gene expression, a similar protocol was carried out for qRT-PCR. HWJSC spheroids were generated as described in Materials and Methods and cultured for 7 total days. After day 7 of spheroid culture, HWJSCs from a single well (300 spheroids) were harvested, washed in DPBS, and cultured in CnT-PR-CC in non-adherent orbital shaker culture for 24 hours. On the same day of HWJSC spheroid harvest, HPEKp were passaged and seeded on polystyrene enhanced attachment microcarrier beads (Corning) at post-confluence (~200,000 cells/cm^2^) by incubating HPEKp with approximately 600 microcarriers in a single well of a 96 well ultra low attachment dish for 6 hours in CnT-PR-CC and subsequently transferring microcarriers to a 6-well ultra low attachment plate with an additional 1 mL of CnT-PR-CC for an additional 18 hours. After both cells and spheroids were cultured for 24 hours in CnT-PR-CC, spheroids and microcarriers were harvested, washed in DPBS, and lysed using Cells-to-CT kit. For microcarriers, lysis was performed by freeze/thawing on dry ice 3 times and subsequently performing DNase digest and transferring to -20°C storage. For HWJSC spheroids, lysis was performed by freeze/thawing three times on dry ice followed by mortar and pestle homogenization for 3 separate 40 second periods, with a 5 min centrifugation at 12,000 x g in between homogenizing cycles. After all samples were collected and lysed (from 3 independent experiments using 600 microcarriers or 300 HWJSC spheroids), standard Cells-to-CT protocol was followed, and sample lysates were analyzed with polymerase chain reaction (PCR) on an ABI thermocycler as described in Materials and Methods and in the Supplementary Methods section above. The C_T_ values were generated using the built-in software, C_T_ values were double normalized to *GAPDH* for both HWJSC samples and HPEKp samples, and the data were double normalized to HPEKp cultures. Statistical analysis was performed using a one-tailed t-test comparing each fold change to a mean value of ‘1’, with a significance level of α=0.05 for three biological replicates per condition.

**Harvesting HWJSC Spheroids for 2D Differential Gel Electrophoresis (2D-DIGE)**

HWJSC spheroids (day 1 or day 7 in culture) from three biological replicate experiments were harvested (3-4 wells per sample), washed in DPBS three times, and lysed in 200 µL of buffer containing 2% SDS, 30 mM Tris, 5 mM DTT. Samples were homogenized with 1 mm diameter Zirconia/Silica Beads (Biospec) for 20 seconds at level 6 on a FastPrep24 homogenizer (MPBiomedicals). Lysates were immediately heat denatured at 95°C for 10 min and centrifuged at 12,000 x g for 1 min. Supernatants were collected and sonicated using a sonic probe for 6 pulses, centrifuged at 14,000 x g for 10 min (at 4°C), transferred to a new centrifuge tube, and stored at -20°C. Samples were precipitated by diluting sample three-fold in 20% trichloroacetic acid on ice for 15 minutes and were centrifuged at 18,000 x g for 10 min at 4°C. The pellet was washed in 10% trichloroacetic acid, centrifuged, washed in ice cold acetone, air dried, dissolved in 7 M urea, 2 M thiourea, 30 mM Tris pH 8.5, 2% CHAPS, and 1% nonidet P-40, and aliquoted and stored at -20°C. Total protein concentration was determined using QuickStart Bradford (Bio-Rad). Samples were labeled with Cy-Dye DIGE Fluor Minimal Labeling Kit (GE Life Sciences) using 35 μg of each protein sample and 280 pmol of either Cy3, Cy5, or Cy2. Cy3 and Cy5 samples were randomized by gel and dye; Cy2 internal reference standard was made by pooling all replicates. Labeled samples were mixed with rehydration buffer (7 M urea, 2 M thiourea, 2% CHAPS, 0.5% IPG Buffer, 0.002% bromophenol blue, 20 mM DTT) and loaded onto pH 3–11 nonlinear IPG strip (GE Life Sciences). IPG strips were actively rehydrated overnight (12.5 hours) at 30V, followed by 500 V hold for 1 h, 1000 V gradient for 1 h, 6000V gradient for 2 h, and 6000V hold for 30 min for a total of 11.6 kVhrs focusing. Proteins in the IPG strips were reduced and alkylated by incubation in equilibration buffer (6 M urea, 75 mM Tris-HCl pH 8.8, 29.3% glycerol, 2% SDS, 0.002% bromophenol blue) containing 10 mg/mL DTT for 15 min followed by incubation in equilibration buffer with 25 mg/mL iodoacetamide. IPG strips were then loaded onto 10% Criterion Pre-cast gels (IPG+1, 1.0 mm; Bio-Rad), sealed with 0.5% agarose overlay, and run at 200V for 1 h. Gels were scanned on a PharosFX Molecular Imager System (Bio-Rad). Images were analyzed using SameSpots (TotalLab), and spots that were differentially expressed by greater than 20% fold change (day 7 relative to day 1) and ANOVA *p*-value < 0.055 were picked for identification with mass spectrometry.

**Immunofluorescence Staining of Day 1 and 7 HWJSC Spheroids**

Day 1 and day 7 HWJSC spheroids were harvested with DPBS and fixed in 4% paraformaldehyde, 1% Triton X-100 (Fisher) in DPBS (pH 7.3, prepared fresh) for 4 hours rotating at 4°C. Spheroids were treated by a graded series of methanol in DPBS (25%, 50%, 75%, 95%) for 30 min at each methanol concentration followed by centrifugation at 3,000 x g for 1 min after each step, discarding of the supernatant, and finally a 3 h incubation in 100% methanol rotating at 4°C. The reverse was performed, and spheroids were subsequently washed in DPBS-T (with Ca^2+^, Mg^2+^, 0.1% Triton X-100) and incubated in 3% BSA (Sigma) in DPBS-T rotating overnight at 4°C. HWJSC spheroids were divided into aliquots, incubated in 1% BSA in DPBS-T for 30 min, and centrifuged to remove supernatant. HWJSC spheroids were incubated in a 1:100 dilution of AlexaFluor 488-tagged antibodies in 1% BSA in DPBS-T rotating for 72 h at 4°C. HWJSC spheroids (Day 1 and Day 7) were stained with polyclonal rabbit anti-human collagen I (Bioss bs10423R-A488), polyclonal rabbit anti-human collagen IV (Bioss bs4595R-A488), polyclonal rabbit anti-human laminin (Novus NB300-144AF488), monoclonal rabbit anti-human fibronectin F1 (Abcam ab198933), or control antibody, polyclonal rabbit anti-goat IgG (Abcam ab150141), which showed no staining on confocal (images not shown). HWJSC spheroids were washed three times in DPBS-T (last wash was for 2 h rotating at 4°C) and incubated in Hoechst 33258 (Life Technologies) at 10 μg/mL in DPBS for 6 h rotating at 4°C. HWJSC spheroids were washed twice in DPBS-T (last wash was for 4 h rotating at 4°C) and were cleared using the Clear^T2^ protocol[[16](#_ENREF_16)]. Spheroids were incubated in 25% formamide (Sigma) and 10% poly(ethylene glycol) 8,000 (Sigma) in DPBS for 20 min at room temperature, segregated by centrifugation, and incubated in 50% formamide and 20% PEG_8000_ in DPBS for at least 20 min. Spheroids in 50% formamide/20% PEG were transferred to optical 96 well plate and imaged using Nikon A1 confocal laser scanning microscope at 10x (with 1.5x magnification) and z-stacks of 3 μm step, 110 μm range.

**Analytical Determination of Dexamethasone Presence and Abundance in OM**

We sought to determine quantitatively the presence of dexamethasone in the osteo-induction (OM) medium to support our hypothesis that dexamethasone in the OM could have elevated PPARG expression in HWJSC spheroids consistent with prior literature. To perform the qualitative identification of the unknown, the product ion spectrum of a dexamethasone (Cayman Chemical) standard was compared to a 70:30 mixture of the OM with acetonitrile by using electrospray ionization in positive ion mode on an AB Sciex (Framingham, MA) 4000 Qtrap linear ion trap mass spectrometer. Chromatographic separation was achieved with a Phenomenex (Torrance, CA) Kinetex XB-C18 (150 x 2.1 mm, 2.6 u) column. The mobile phases were 0.1% formic acid (A) and 0.1% formic acid in acetonitrile (B). The flow rate was 400 uL/min. The collision energy for the product ion spectra was 17 V. Quantitation of the unknown was performed on the same system using multiple reaction monitoring (MRM) mode with collision energy optimized for each transition. The unknown was quantitated against an external calibration curve of dexamethasone that used 5 points over a range of 1 – 50 ng/mL. All solvents were LC/MS grade. Solvents and formic acid were purchased from Thermo Fisher Scientific. The correlation coefficient (r) for the calibration curve was 0.993. All points on the curve were within 20% of the actual amount. For product ion scans and in MRM mode, the retention time of the ion of m/z 393.2 in the cell media matched the retention time of the molecular ion (m/z 393.2) of the dexamethasone standard. The retention time window observed for dexamethasone standards was 1.93-1.99 min. The retention time observed for the ion of m/z 393.2 in triplicate analysis of the cell media was 1.97 ± 0.02 min. The product ion spectra obtained for the unknown and the standard were highly similar. See Supplemental Table 6 and Supplemental Fig. 7.

For quantitation of the abundance of dexamethasone in the OM, the MRM transition of m/z 393.2 to m/z 373.1 was monitored for the dexamethasone standard and a 50:50 mixture of the cell media with acetonitrile. Two additional MRM transitions were monitored for confirmation: m/z 393.2 to m/z 355.1; and m/z 393.2 to m/z 237.1. The amount of dexamethasone observed for triplicate measurements in cell media was 24.2 ± 1.02 ng/mL. Since the MRM transitions are characteristic for a species, the area ratios of the transitions can be used to confirm identify of compounds. Supplemental Table 7 gives the observed area ratio ranges for the quantifier transition to each of the confirmation transitions, as observed for the dexamethasone standards. The same transitions were monitored for betamethasone, an isomer of dexamethasone. Comparison of the average transition area ratio ranges observed for triplicate analysis of the sample mixture indicates agreement with the expected ratio ranges for dexamethasone. The ratio ranges for the cell media ion are not consistent with those observed for betamethasone. The retention time agreement, similarity of the product ion scans, and overlap of the observed MRM area ratios with the acceptance range for dexamethasone standards support identification of the ion of m/z 393.2 in the cell media as dexamethasone. The retention time agreement, similarity of the product ion scans, and overlap of the observed MRM area ratios with the acceptance range for dexamethasone standards support identification of the ion of m/z 393.2 in the cell media as dexamethasone. The amount of dexamethasone observed for triplicate measurements in cell media was 24.2 ± 1.02 ng/mL.
